# Supplementary figures and images for: Validation of FUNMOVES: A reliable tool for assessing motor skills in Spanish schoolchildren
Source: PLoS One. 2025 Dec 5;20(12):e0337605. doi: 10.1371/journal.pone.0337605 (PMC12680221; doi:10.1371/journal.pone.0337605)

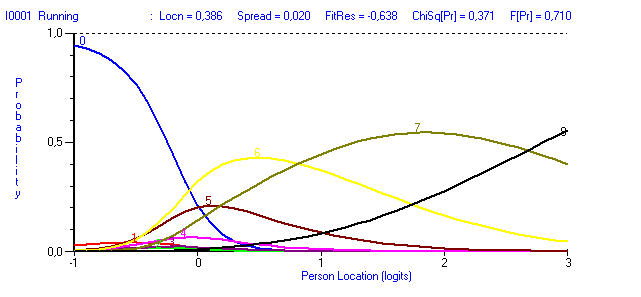

Supplement: S1 Fig — Fig2. a1) Running. (TIF) [file pone.0337605.s001.tif]

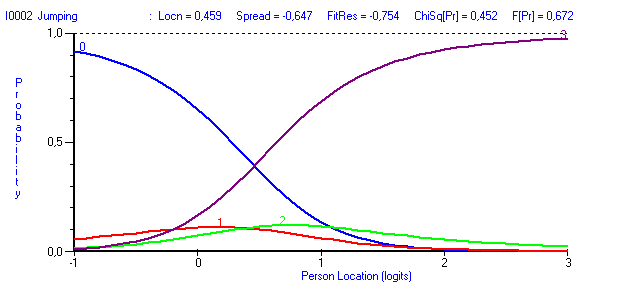

Supplement: S2 Fig — Fig2. a2) Jumping. (TIF) [file pone.0337605.s002.tif]

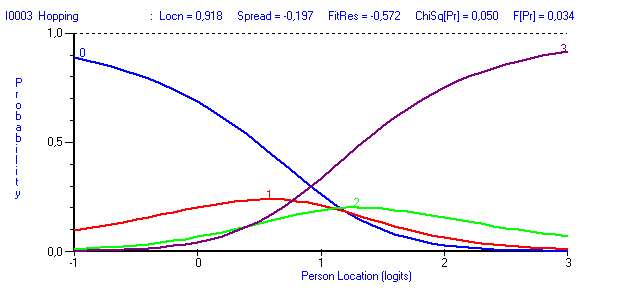

Supplement: S3 Fig — Fig2. a3) Hopping. (TIF) [file pone.0337605.s003.tif]

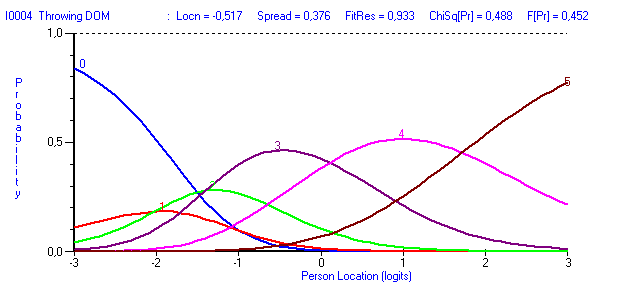

Supplement: S4 Fig — Fig2. a4) Throwing DOM. (TIF) [file pone.0337605.s004.tif]

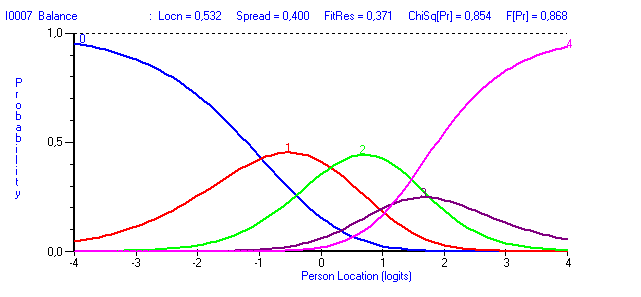

Supplement: S5 Fig — Fig2. a5) Balance. (TIF) [file pone.0337605.s005.tif]

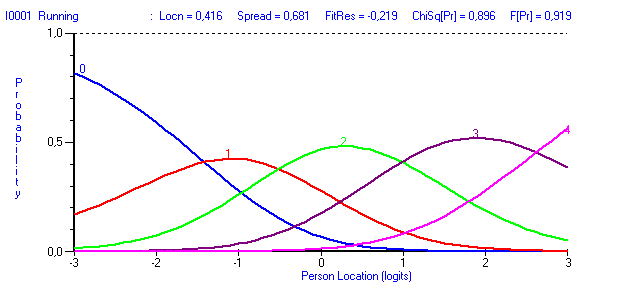

Supplement: S6 Fig — Fig2. b1) Running rescored. (TIF) [file pone.0337605.s006.tif]

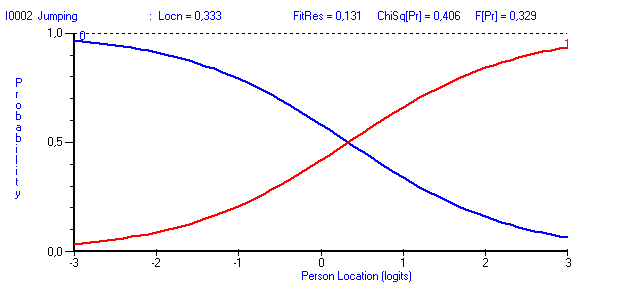

Supplement: S7 Fig — Fig2. b2) Jumping rescored. (TIF) [file pone.0337605.s007.tif]

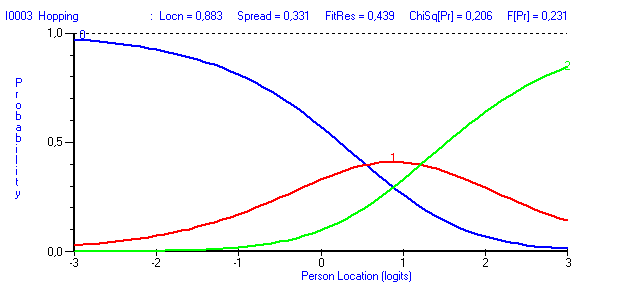

Supplement: S8 Fig — Fig2. b3) Hopping rescored. (TIF) [file pone.0337605.s008.tif]

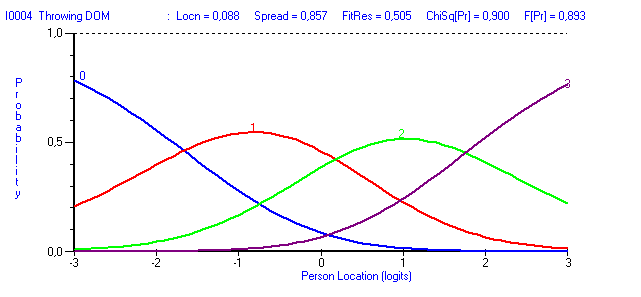

Supplement: S9 Fig — Fig2. b4) Throwing rescored. (TIF) [file pone.0337605.s009.tif]

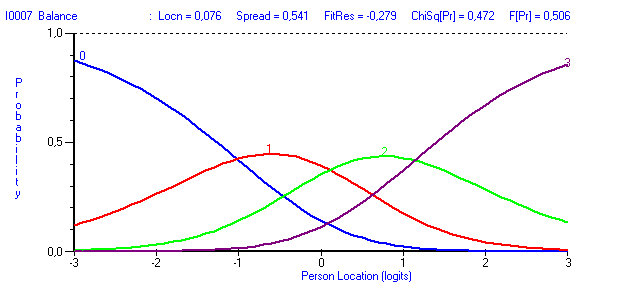

Supplement: S10 Fig — Fig2. b5) Balance rescored. (TIF) [file pone.0337605.s010.tif]

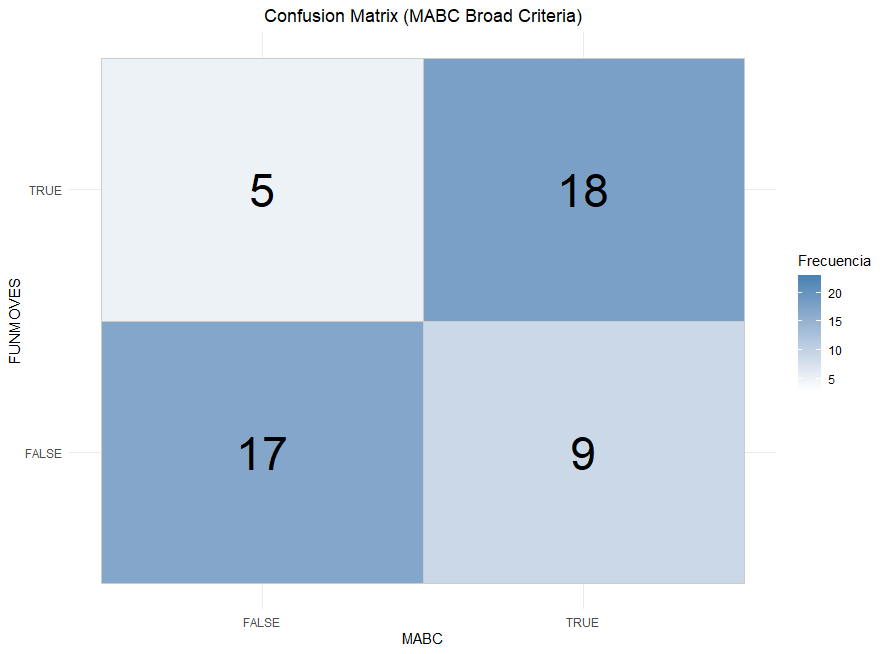

Supplement: S11 Fig — (TIF) [file pone.0337605.s011.tif]

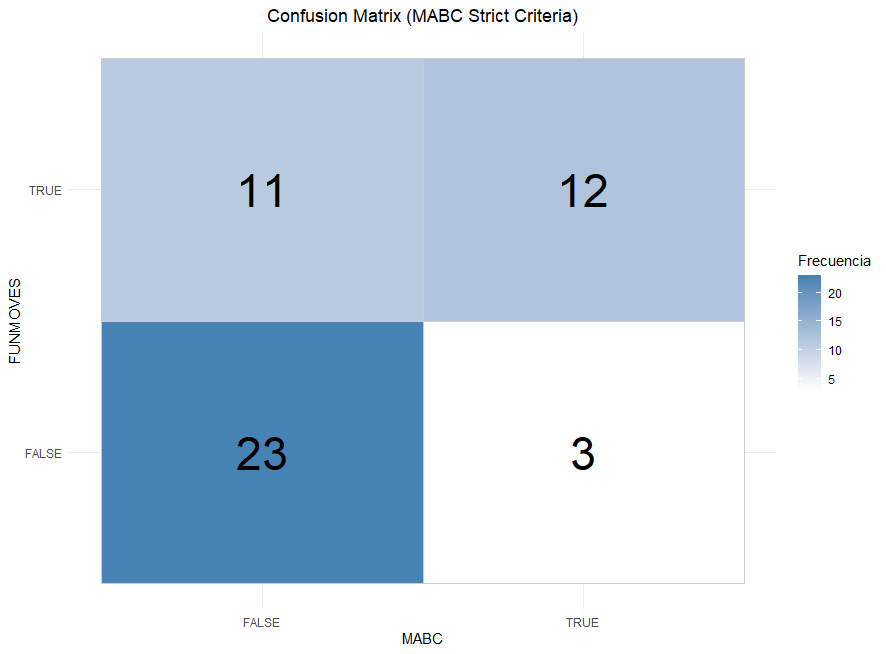

Supplement: S12 Fig — (TIF) [file pone.0337605.s012.tif]

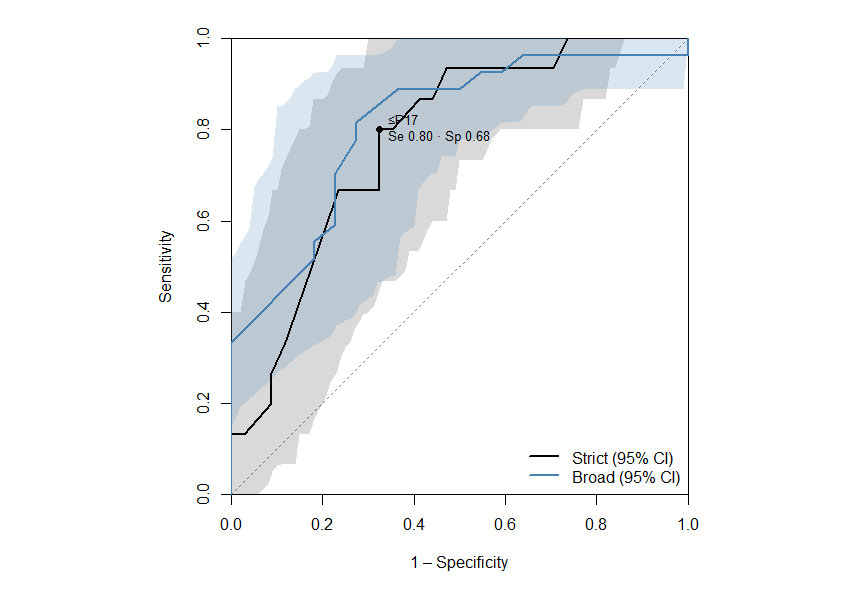

Supplement: S13 Fig — (TIF) [file pone.0337605.s013.tif]
