# Supplementary material for: Validation of FUNMOVES: A reliable tool for assessing motor skills in Spanish schoolchildren
Source: PLoS One. 2025 Dec 5;20(12):e0337605. doi: 10.1371/journal.pone.0337605 (PMC12680221; doi:10.1371/journal.pone.0337605)
Supplement: S1 File — (PDF) [file pone.0337605.s014.pdf]

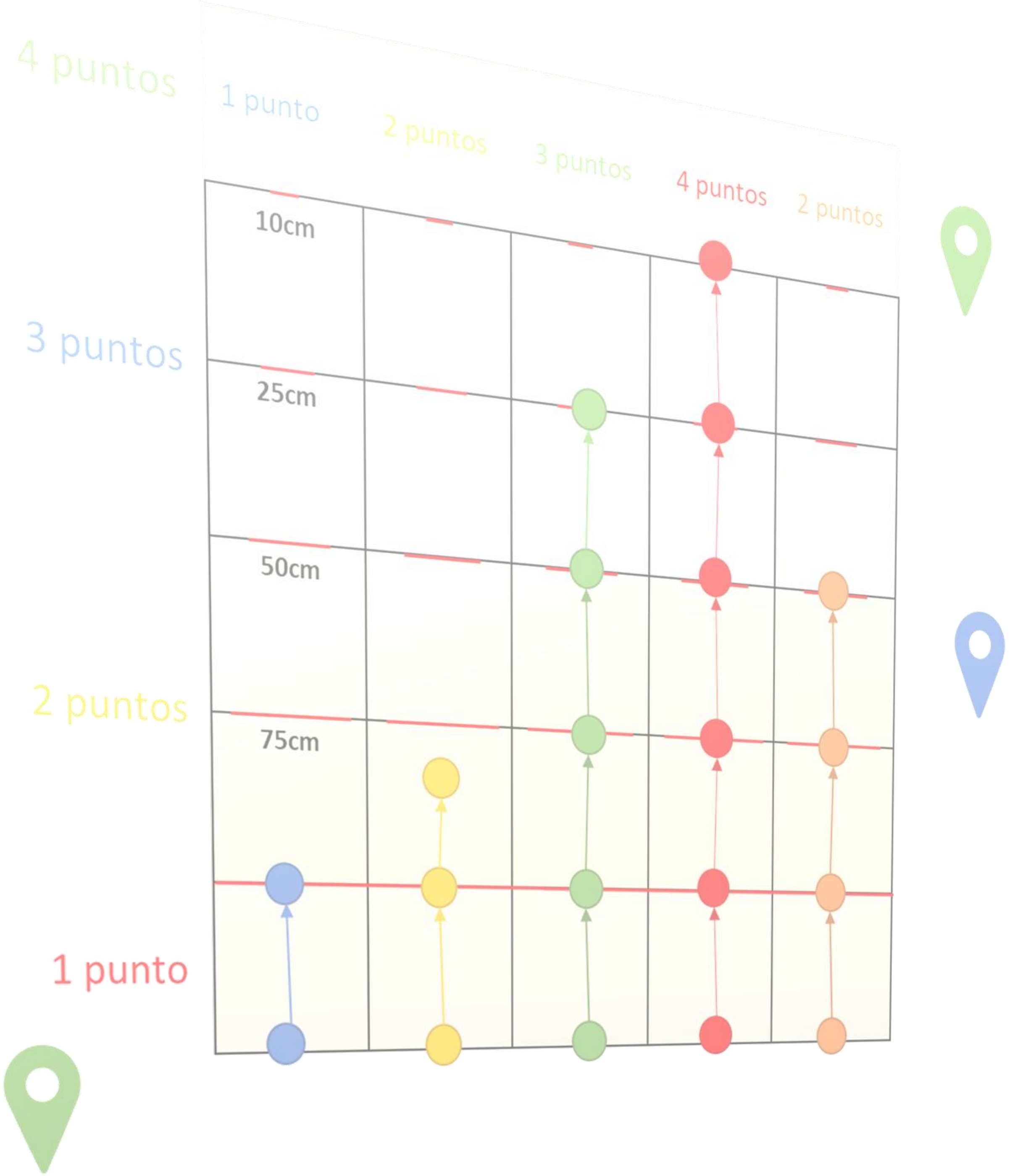

FUNMOVES – ADAPTACIÓN ESPAÑOLA

UNA GUÍA DEL USUARIO PARA CONFIGURAR, EJECUTAR Y PUNTUAR LAS ACTIVIDADES

Universidad de Navarra | FACULTAD DE EDUCACIÓN Y PSICOLOGÍA

UNIVERSITY OF LEEDS

I. Introducción ______ ***2***

Objetivo de FUNMOVES ______ **2**

¿Qué es FUNMOVES? ______ **2**

¿Para qué se utiliza FUMOVES? ______ **3**

II. Fases ______ ***4***

1. Preparación de la prueba ______ **4**

Recursos necesarios ______ 4

Grupos de clase ______ 4

Marcar la rejilla de FUNMOVES ______ 5

Implementación de FUNMOVES ______ 6

2. Pruebas ______ **7**

Prueba 1: Carrera ______ 8

Prueba 2: Saltos con los pies juntos ______ 10

Prueba 3: Saltos con un solo pie ______ 13

Prueba 4: Lanzamientos ______ 16

Prueba 5: Golpeo con el pie ______ 18

Prueba 6: Equilibrios ______ 20

3. Recogida de datos ______ **23**

I. Introducción

Este manual ha sido adaptado por el grupo de investigación 'Neurodesarrollo y Aprendizaje' de la Universidad de Navarra a partir del instrumento original FUNMOVES desarrollado por la Universidad de Leeds.

Se publica bajo una licencia 'Creative Commons Atribución' (CC BY), lo que permite su uso, distribución y adaptación sin restricciones, siempre que se cite adecuadamente la fuente original y la adaptación realizada.

Objetivo de FUNMOVES

El objetivo principal de FUNMOVES es **proporcionar a las escuelas una herramienta eficaz para evaluar las habilidades fundamentales del movimiento en los niños/as.** Al identificar a los niños/as que tienen dificultades con las habilidades motrices claves, se puede ofrecer un apoyo adicional y personalizado para ayudarles a mejorar sus habilidades y reducir el riesgo de problemas de salud física y mental, así como un bajo rendimiento académico. Además, FUNMOVES ha sido diseñada pensando en la factibilidad de su uso en las escuelas, lo que significa que puede evaluarse a toda una clase en una hora durante la asignatura de Educación Física.

¿Qué es FUNMOVES?

FUNMOVES es una **herramienta de evaluación** basada en la evidencia diseñada en la Universidad de Leeds por parte del equipo de investigación compuesto por: Lucy Eddy, dirigida por Liam Hill y Nick Preston para poder identificar posibles dificultades en **habilidades fundamentales del movimiento (HFM)** en niños/as. Se centra específicamente en seis habilidades motoras claves: correr, saltar, saltar con un solo pie, lanzar, golpear con el pie y mantener el equilibrio. Como es una herramienta de 'screening', el resultado de la evaluación con FUNMOVES es un apoyo extra a la información que tengan en el centro y la familia que permita apoyarse en un instrumento de evaluación para la sospecha de posibles dificultades en HFM.

Las pruebas de FUNMOVES se desarrollan en una **rejilla de 5x5 metros** donde cada alumno tiene su propio carril de 1 metro de ancho por 1 metro de largo. Durante la evaluación, 5 niños/as participan al mismo tiempo en las diferentes habilidades motrices antes mencionadas. La rejilla proporciona un espacio de evaluación estandarizado que asegura que cada niño tenga suficiente espacio para realizar las habilidades requeridas.

Al evaluar a **5 niños/as simultáneamente**, se puede completar la evaluación de toda la clase en una hora durante la asignatura de educación física. Esta forma eficiente de evaluación permite que los profesores/as puedan centrarse en desarrollar planes de enseñanza individualizados para cada niño/a en función de los resultados de la evaluación.

Además, es importante mencionar que los **materiales** utilizados en la evaluación de FUNMOVES son de **bajo coste y de uso cotidiano** en la asignatura de Educación Física. Esto significa que el centro no tendrá que hacer una inversión significativa para utilizar la prueba, ya que los materiales necesarios, como sacos, cintas de marcaje y cronómetro, son fácilmente accesibles y se utilizan regularmente en la clase de educación física. Esto permite que las escuelas de diferentes presupuestos puedan utilizar FUNMOVES para identificar las habilidades motoras de sus estudiantes y proporcionar el apoyo adicional necesario sin incurrir en costos adicionales.

¿Para qué se utiliza FUMOVES?

La investigación ha mostrado que los niños con bajas habilidades fundamentales del movimiento tienen un **mayor riesgo** de sufrir consecuencias adversas en la infancia, como **problemas de salud física y mental**, así como un **bajo rendimiento académico**. Identificar a los niños/as que tienen dificultades con las habilidades motrices claves ayudará a las escuelas a dirigir el apoyo de forma eficaz a dicho alumnado.

FUNMOVES permite por tanto a los maestros/as de Educación Física poder colaborar con los maestros/as de otras asignaturas, así como con el departamento de orientación de cada centro para poder entender el origen de las dificultades de algunos niños dentro del aula. Por tanto, los resultados de esta evaluación permitirán conocer más datos del desempeño del niño/a y poder sospechar de dificultades que lastren la fluidez de su aprendizaje.

II. Fases

1. Preparación de la prueba

Recursos necesarios

Rejilla

- ✓ 25 bolsas de alubias
- ✓ 60 m cinta de marcar negra
- ✓ 15 m cinta de marcar color

Carpeta del maestro/a

- ✓ Cronómetro
- ✓ Hojas de puntuación
- ✓ Bolígrafo
- ✓ Pizarra zonas + rotulador
- ✓ Ayuda de otro profesional del centro para puntuar las actividades

Grupos de clase

1. Separe su clase en grupos de hasta cinco, según la capacidad de los niños/as

Los grupos de niños/as pueden ser de hasta 5 personas teniendo en cuenta la dificultad para poder observar a todos/as al mismo tiempo y la duración de la clase. A la hora de establecer los grupos, tenga en cuenta la habilidad de cada niño/a para correr, saltar, saltar con un solo pie, lanzar, golpear con el pie y mantener el equilibrio. Estos niños/as participarán al mismo tiempo cada uno en uno de los carriles de la rejilla. El hecho de que los niños/as con las mismas habilidades participen a la vez responde a dos objetivos. En primer lugar: que todos alcancen un resultado parecido provocando que no existan comparaciones. En segundo lugar: que su desempeño no quede condicionado por sus pares, es decir, que el niño/a no tenga referencias simultaneas de gente que en principio lo va a hacer mejor o peor que el/ella.

2. Complete la información demográfica en la hoja de puntuación (para el maestro)

La mano dominante debe anotarse como la mano con la que el niño/a escribe.

Debe indicar si cree que un niño/a: tiene dificultad motriz, si tiene dificultades para escribir a mano, si es torpe cuando se mueve por el aula, o si tiene dificultades para interactuar físicamente con los objetos.

CLASE ____________________

	Nombre 1	Nombre 2	Nombre 3	Nombre 4	Nombre 5
**Información demográfica**					
Sexo					
Fecha de nacimiento					
Mano dominante					
¿Crees que este niño tiene dificultad motriz?					

Marcar la rejilla de FUNMOVES

Todas las actividades se desarrollan en una zona cuadriculada de 5x5 metros, que debe establecerse utilizando cinta y con la ayuda de una cinta métrica para las especificaciones que se muestran a continuación. Hay que tener en cuenta que las líneas de color rojo del diagrama deben estar marcadas en un color diferente al color del resto de la cuadrícula. Por último, es necesario asegurarse de que hay suficiente sitio en el aula para que los niños/as que no participan en la prueba en ese momento puedan permanecer sentados.

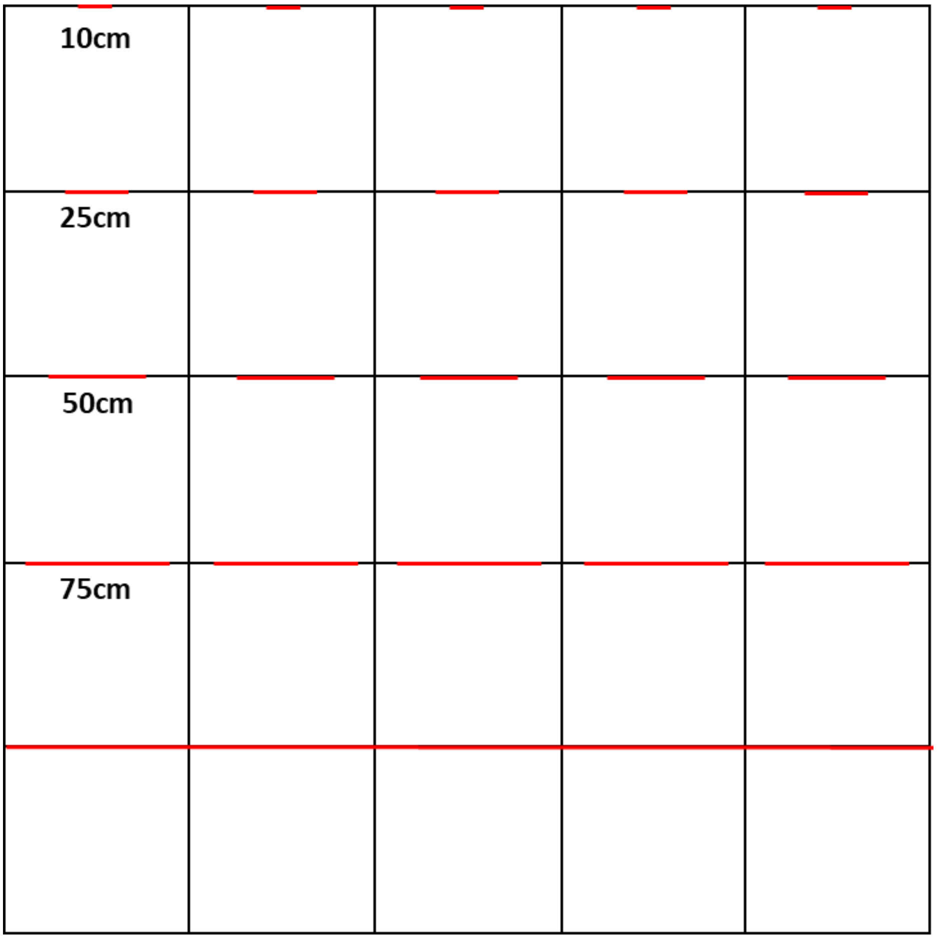

Implementación de FUNMOVES

FUNMOVES puede implementarse como una prueba de evaluación o también puede llevarse a cabo como una competición por equipos para que sea divertida y atractiva.

- ✓ Durante la prueba, pida a los niños/as que no están participando de la tarea en ese mismo momento, que esperen en un banco.
- ✓ Realice las actividades de una en una, evaluando a todos los niños/as antes de pasar a la siguiente actividad.
- ✓ No permita que los niños/as practiquen una tarea antes de realizarla en la prueba.
- ✓ Para evitar que los niños/as, no completen las tareas correctamente, dígales que no recibirán ningún punto para su equipo si hacen trampa. No tienen que sentirse evaluados, pero sí que lo hagan lo mejor posible.
- ✓ Intente no hacer evidente cuando un niño/a ha cometido un error o si va "ganando".
- ✓ Anote los intentos fallidos en la hoja de puntuación, pero permita que los niños/as continúen y completen todas las actividades, independientemente del nivel alcanzado.
- ✓ Realizar los grupos de 5 participantes con anterioridad y distribuirlos en grupos teniendo cada uno del grupo un carril asignado.
- ✓ Llevar las hojas de registro cumplimentadas con la parte de información personal atendiendo a los grupos realizados.
- ✓ Junto con este manual, existe disponible una presentación de 'Power Point', así como unos videos explicativos de cada prueba que complementan la información presente en este manual de una manera más gráfica.

2. Pruebas

Todas las pruebas que se realizan en FUNMOVES tienen lugar en el espacio delimitado por la rejilla de 5 x 5 metros. En ella cada alumno/a tendrá su propio carril para realizar cada una de las tareas siendo cada una de las marcas necesarias para las distintas pruebas.

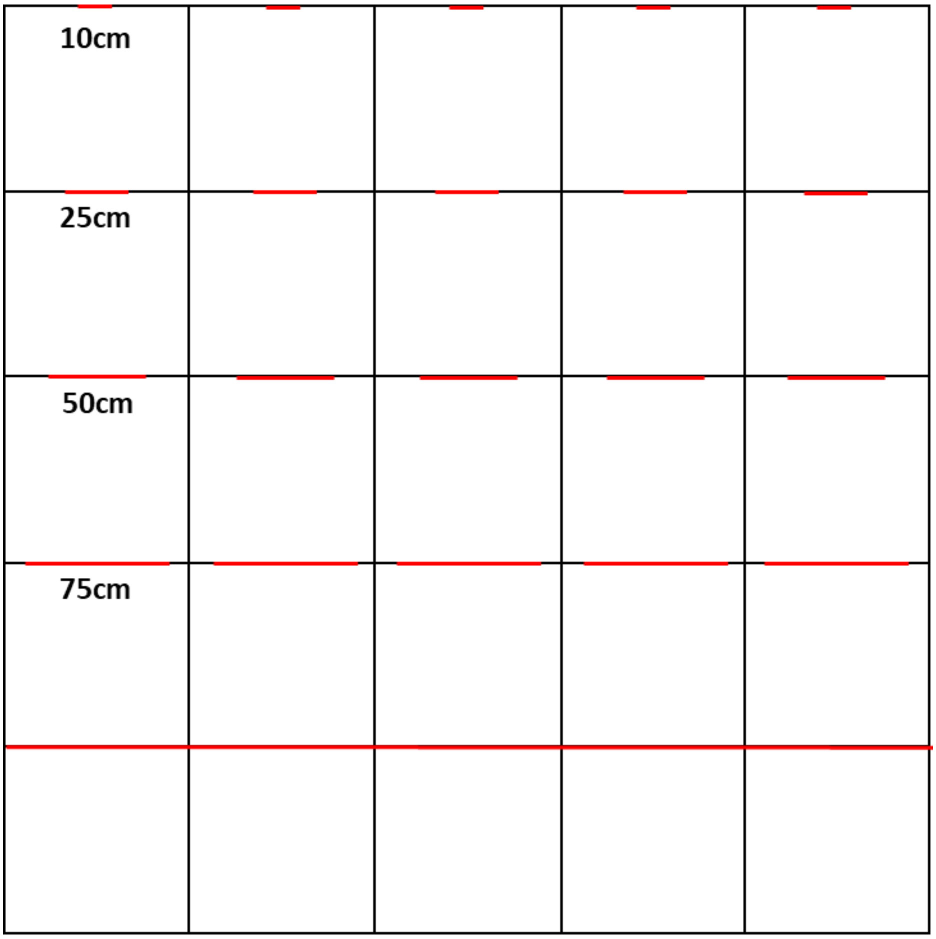

Prueba 1: Carrera

La prueba de carrera consiste en cubrir el mayor número de veces la distancia de 5 metros durante 15 segundos. Para ello el niño/a partirá con los dos pies pisando la línea que marca el inicio de su carril y al recibir la orden "3, 2, 1, ya" saldrá corriendo hasta alcanzar la última de las líneas de su carril para después volver al punto de partida. Este proceso deberá hacerlo el mayor número de veces hasta que a la voz de "tiempo" se tendrá que detener. La puntuación de dicha prueba será el número de largos completos (5m) que se hayan efectuado.

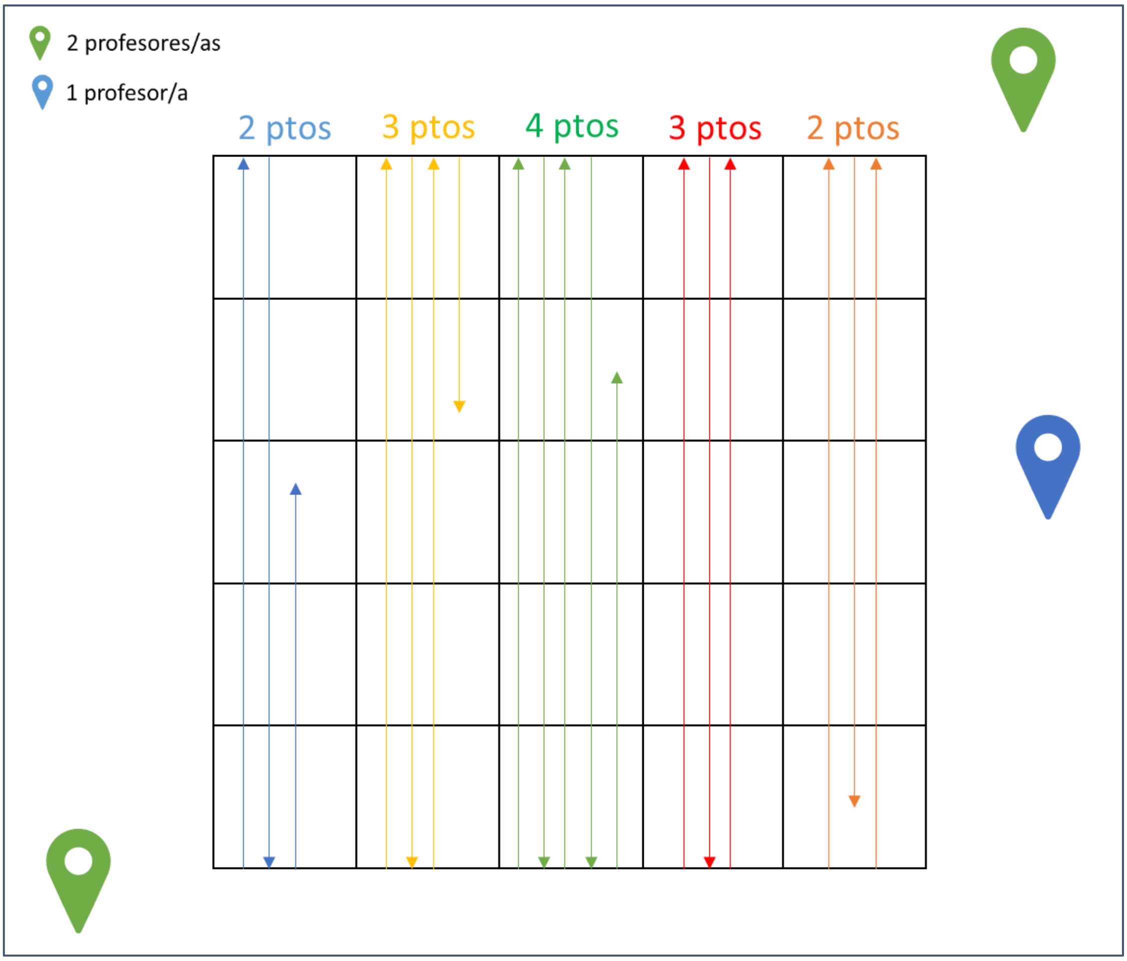

Ilustración 1 - Gráfico de ejemplos de la prueba 1: carrera

Reglas e instrucciones para comunicar a los niños/as

- ✓ Objetivo: **mayor número de largos** en el tiempo marcado.
- ✓ Tiempo límite: **15 segundos**.
- ✓ Fin de la prueba: al escuchar la palabra "tiempo" detenerse/sentarse hasta ser avisado.
- ✓ Punto clave 1: correr por el **propio carril**. Avisar si alguien se sale.

- ✓ Punto clave 2: tanto en la línea final como en la inicial y en cada uno de los largos hay que **pisar con claridad la línea** al menos con uno de los pies.
- ✓ Punto clave 3: a pesar de que se puede **deslizar con el pie**, no es una buena estrategia puesto que se pierde tiempo.
- ✓ Punto clave 4: después de realizar la demostración poner ejemplos de los posibles fallos en la prueba. Explicar qué cosas no se pueden hacer.

Puntuación de la prueba y errores más comunes

- ✓ Puntuación: **número total de largos (5m) completos**.
- ✓ Error no sancionable: **salirse del carril**. Hay que avisar que ocupe el carril propio.
- ✓ Error sancionable: **no pisar la línea** al realizar un largo. Este largo no contará para el cómputo final de puntos. Hay que avisar de que no se ha pisado.
- ✓ Error sancionable: **no realizar un cambio de sentido** al llegar al final, sino hacer una pequeña curva. Hay que avisar de que no se ha hecho cambio de sentido.

Prueba 2: Saltos con los pies juntos

La prueba de saltar con los pies juntos consiste en ir dando pequeños saltos hasta alcanzar la siguiente marca de aterrizaje, una vez ahí realizar una pausa, sin perder el equilibrio y sin mover los pies, para esperar a que se indique el inicio o se dé la señal de salida. Repetir los pasos sucesivamente con cada una de las marcas del carril propio. En este caso no se recibirán puntos por hacerlo rápido sino por hacerlo sin perder el equilibrio en ningún momento de la prueba. Según la zona donde el niño/a pierda el equilibrio recibirá una cantidad de puntos u otra.

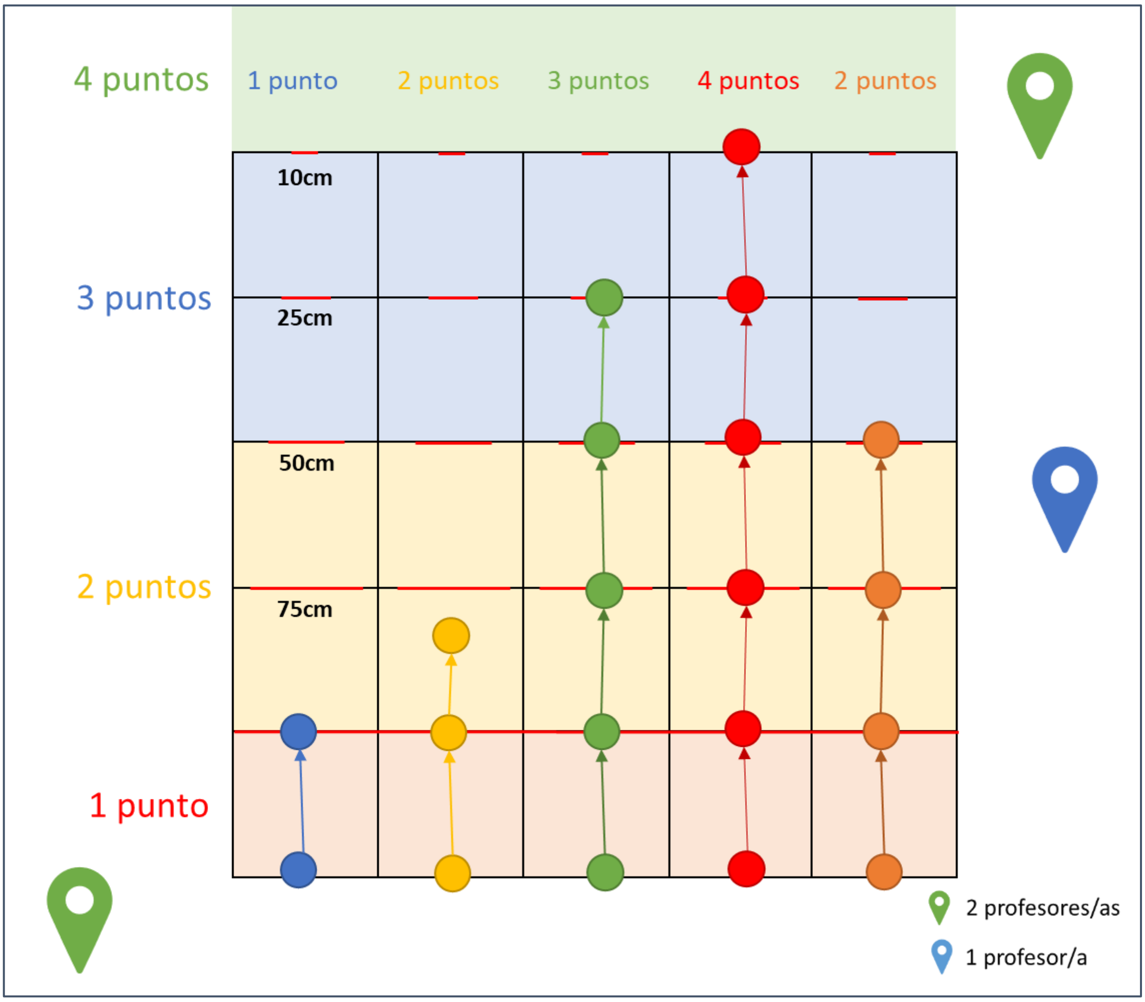

Ilustración 2 - Gráfico de ejemplos de la prueba 2: saltos pies juntos

Reglas e instrucciones para comunicar a los niños/as

- ✓ Objetivo: avanzar con **pequeños saltitos con pies juntos hasta** realizar una pausa en cada una de **las marcas manteniendo el equilibrio**.
- ✓ **Zonas de pausa**: en cada una de las marcas los niños/as deberán esperar a que lleguen el resto de compañeros/as sin perder el equilibrio y no salir hacia la siguiente marca hasta que el maestro/a diga “1, 2 y 3”.

- ✓ **Fin de la prueba**: cuando el niño/a aguanta los 3 segundos en la marca final. Cuando un niño/a pierde el equilibrio (en este caso registrar el dato y dejar terminar sin llamar la atención).
- ✓ Punto clave 1: las marcas cada vez son más estrechas así que hay que **fijarse en el propio carril** de cada uno para apoyar en ellas.
- ✓ Punto clave 2: los saltos deben ser **pequeños y con pies juntos**. Intentando ocupar el máximo espacio dentro de la marca de aterrizaje.
- ✓ Punto clave 3: realizar las **pausas** (incluida la última) correctamente sin perder el equilibrio.
- ✓ Punto clave 4: después de realizar la demostración poner ejemplos de los posibles fallos en la prueba. Explicar qué cosas no se pueden hacer.

Puntuación de la prueba y errores más comunes

- ✓ Puntuación: **zona donde el niño/a pierde el equilibrio (ver ilustración anterior)**. Si llega al final sin perder el equilibrio, recibirá la máxima puntuación (4 puntos). En el caso de que el equilibrio se pierda en una línea de separación de zonas contará la puntuación de la zona inmediatamente anterior
- ✓ Error 1: realizar **un solo salto** entre marca y marca.
- ✓ Error 2: apoyar un **pie fuera de la marca** teniendo espacio en ella.
- ✓ Error 3: no realizar correctamente la **pausa**. O realizarla en medio del recorrido.
- ✓ Error 4: **perder el equilibrio**. Se explican a continuación del gráfico las situaciones que se consideran o no: perdida de equilibrio.

Cuando en la prueba de saltos con los pies juntos hablamos de perdida de equilibrio la situación más evidente es en la que el niño/a **se caen al suelo**, sin embargo, hay otras situaciones donde el niño/a pierde el equilibrio y su manifestación más clara es el **arrastre del pie**. Un ejemplo ilustrativo es pensar en el movimiento de un flan cuando “tiembla”, que en el terreno de las HFM tendría la denominación de ajuste propioceptivo. Todos estos ajustes donde el niño/a **no desplaza su apoyo serían legales**. En el caso en el que el pie se **arrastre de su apoyo inicial, este se contaría como fallo**.

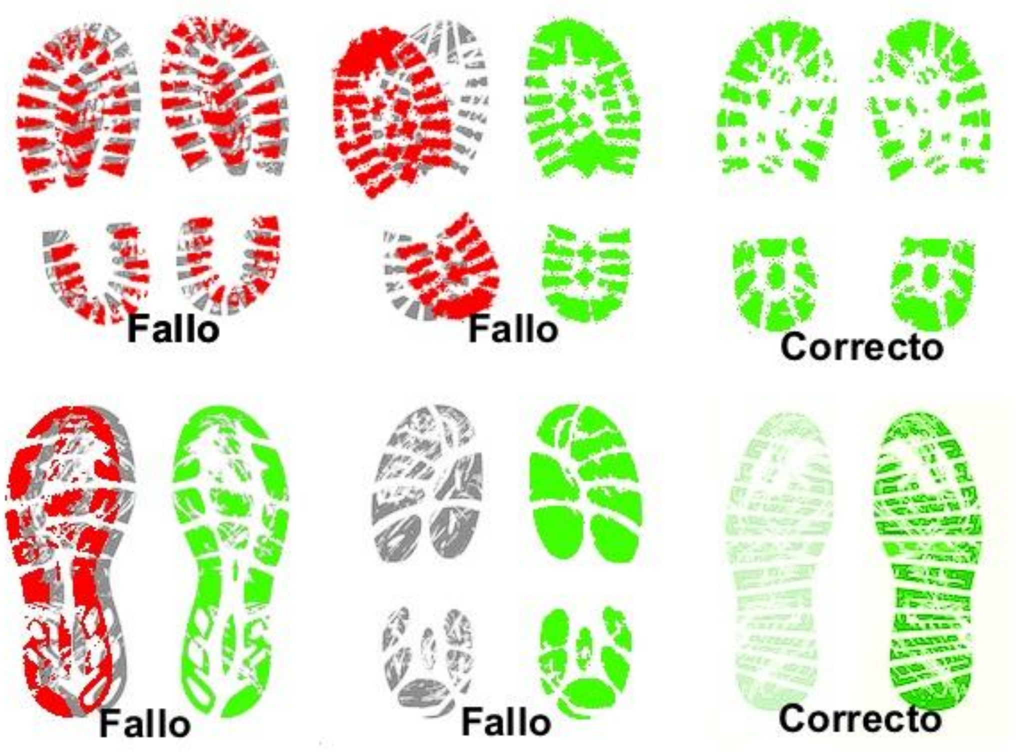

Ilustración 3 - Fallos provocados por la pérdida del equilibrio

Ejemplos de situaciones válidas:

- ✓ Los dos pies están fijos **sin arrastrarse** por el suelo y la huella que realiza el niño/a es fija.
- ✓ **La fuerza recae sobre el apoyo de un pie**, pero el otro no se levanta nunca del suelo.
- ✓ Los pies realizan un balanceo **sobre su propia huella** que termina estabilizándose.

Ejemplos de situaciones incorrectas o de pérdidas de equilibrio:

- ✓ Caerse.
- ✓ Uno o ambos **pies se arrastran** por el suelo modificando su posición inicial en la marca.
- ✓ Uno o ambos **pies se levantan** de su posición inicial en la marca.

Prueba 3: Saltos con un solo pie

La prueba de saltar con un solo pie, es similar a la prueba 2, ya que el único cambio es que se realiza con un solo pie. Esta consiste en ir dando pequeños saltos hasta alcanzar la siguiente marca de aterrizaje, una vez ahí realizar una pausa, sin perder el equilibrio y sin mover el pie, para esperar a que se indique el inicio o se dé la señal de salida. Repetir los pasos sucesivamente con cada una de las marcas del carril propio. En este caso no se recibirán puntos por hacerlo rápido sino por hacerlo sin perder el equilibrio en ningún momento de la prueba. Según la zona donde el niño/a pierda el equilibrio recibirá una cantidad de puntos u otra.

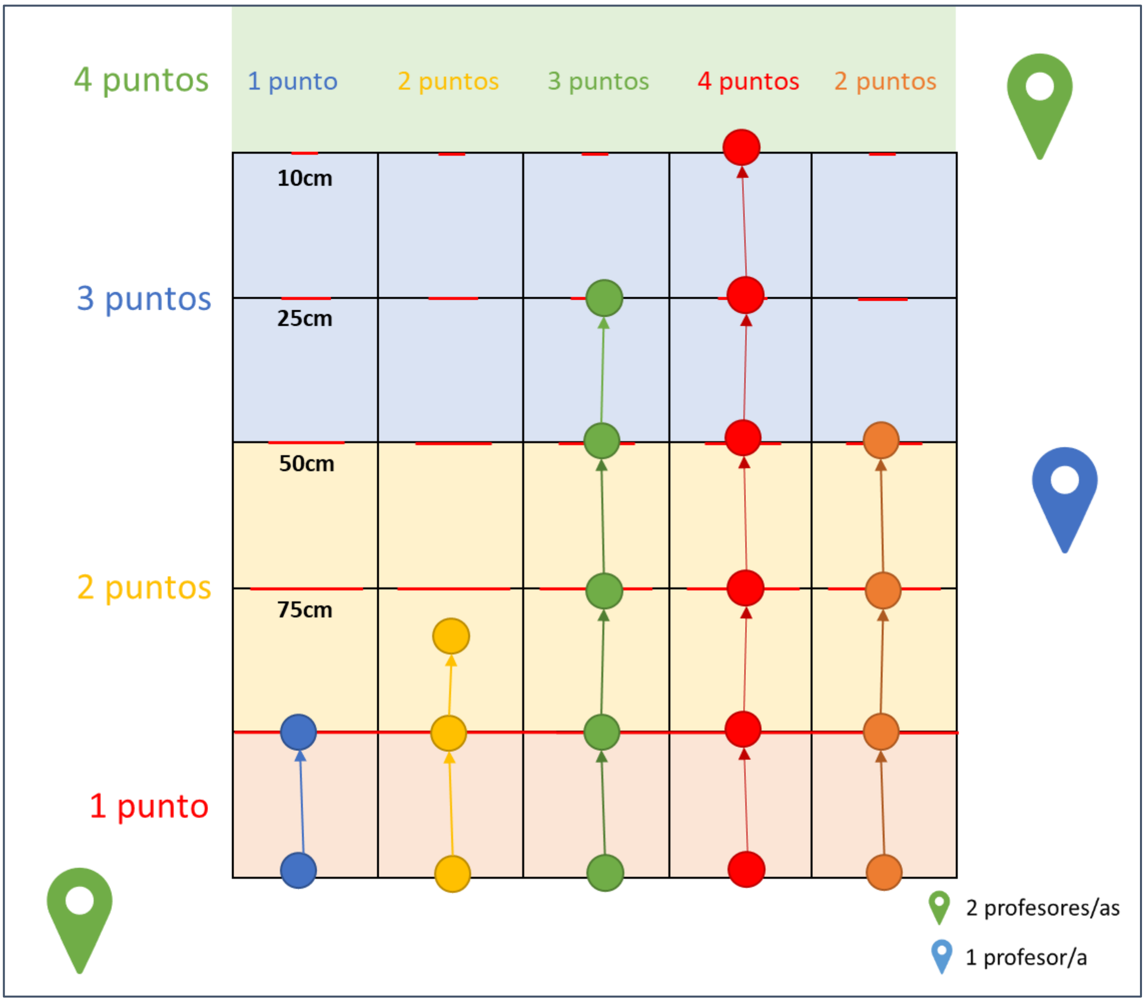

Ilustración 4 - Gráfico de ejemplos de la prueba 3: saltos con un solo pie

Reglas e instrucciones para comunicar a los niños/as

- ✓ Objetivo: avanzar con **pequeños saltitos sobre una pierna hasta** realizar una pausa en cada una de **las marcas manteniendo el equilibrio**.
- ✓ **Elección de la pierna de apoyo**: la pierna de apoyo es a elección del niño/a y no se puede cambiar durante el transcurso de la prueba.

- ✓ **Zonas de pausa**: en cada una de las marcas los niños/as deberán esperar a que lleguen el resto de compañeros/as sin perder el equilibrio y no salir hacia la siguiente marca hasta que el maestro/a diga "1, 2 y 3".
- ✓ **Fin de la prueba**: cuando el niño/a aguanta los 3 segundos en la marca final. Cuando un niño/a pierde el equilibrio (en este caso registrar el dato y dejar terminar sin llamar la atención).
- ✓ Punto clave 1: las marcas cada vez son más estrechas así que hay que **fijarse en el propio carril** de cada uno para apoyar en ellas.
- ✓ Punto clave 2: los saltos deben ser **pequeños y con un solo pie**. Intentando ocupar el máximo espacio dentro de la marca de aterrizaje.
- ✓ Punto clave 3: realizar **las pausas** (incluida la última) correctamente sin perder el equilibrio.
- ✓ Punto clave 4: después de realizar la demostración poner ejemplos de los posibles fallos en la prueba. Explicar qué cosas no se pueden hacer.

Puntuación de la prueba y errores más comunes

- ✓ Puntuación: **zona donde el niño/a pierde el equilibrio**. Si llega al final sin perder el equilibrio, recibirá la máxima puntuación (4 puntos). En el caso de que el equilibrio se pierda en una línea de separación de zonas contará la puntuación de la zona inmediatamente anterior.
- ✓ Error 1: realizar **un solo salto** entre marca y marca.
- ✓ Error 2: apoyar el **pie fuera de la marca** teniendo espacio en ella.
- ✓ Error 3: no realizar correctamente **la pausa**. O realizarla en medio del recorrido.
- ✓ Error 4: **perder el equilibrio**. Se explican a continuación del gráfico las situaciones que se consideran o no: perdida de equilibrio.

Cuando en la prueba de saltos con un solo pie hablamos de perdida de equilibrio la situación más evidente es en la que el niño/a se cae al suelo, sin embargo, hay otras situaciones donde el niño/a pierde el equilibrio y su manifestación más clara es el arrastre del pie. Un ejemplo ilustrativo es pensar en el movimiento de un flan cuando "tiembla", que en el terreno de las HFM tendría la denominación de ajuste propioceptivo. Todos estos ajustes donde el niño/a **no desplaza su apoyo serían legales**. En el caso en el que el pie se **arrastre de su apoyo inicial, este se contaría como fallo**.

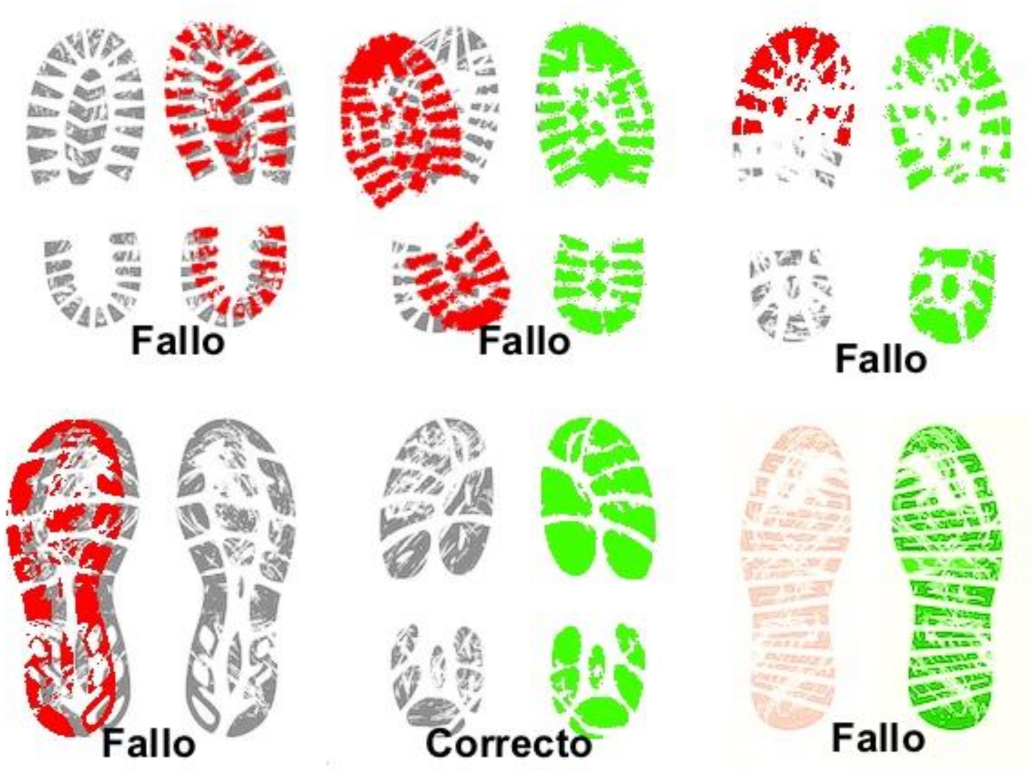

Ilustración 5 - Fallos provocados por la pérdida del equilibrio

Ejemplos de situaciones válidas:

- ✓ El pie de apoyo está fijo **sin arrastrarse** por el suelo y la huella que realiza el niño/a es fija.
- ✓ **La fuerza recae sobre el apoyo de un pie**, y este no se levanta nunca completamente del suelo.
- ✓ El pie de apoyo realiza un balanceo **sobre su propia huella** que termina estabilizándose.

Ejemplos de situaciones incorrectas o de pérdidas de equilibrio:

- ✓ Caerse.
- ✓ **Apoyar el otro pie** parcial o completamente, en el suelo o sobre la otra pierna.
- ✓ El **pie de apoyo se arrastra** por el suelo modificando su posición inicial en la marca.
- ✓ El **pie de apoyo se levanta** completamente de su posición inicial en la marca.

Prueba 4: Lanzamientos

La prueba de lanzamientos consiste en lanzar 5 sacos de uno en uno intentando, dentro del propio carril, dejar cada uno de ellos en un cuadrado. Aquellos sacos que no estén completamente dentro de un cuadrado no contarán. Además, cada cuadrado tendrá una máxima puntuación de un saco, por tanto, si hay dos sacos solo contaría 1 punto. Esta prueba se realizará tanto con la mano derecha como con la mano izquierda.

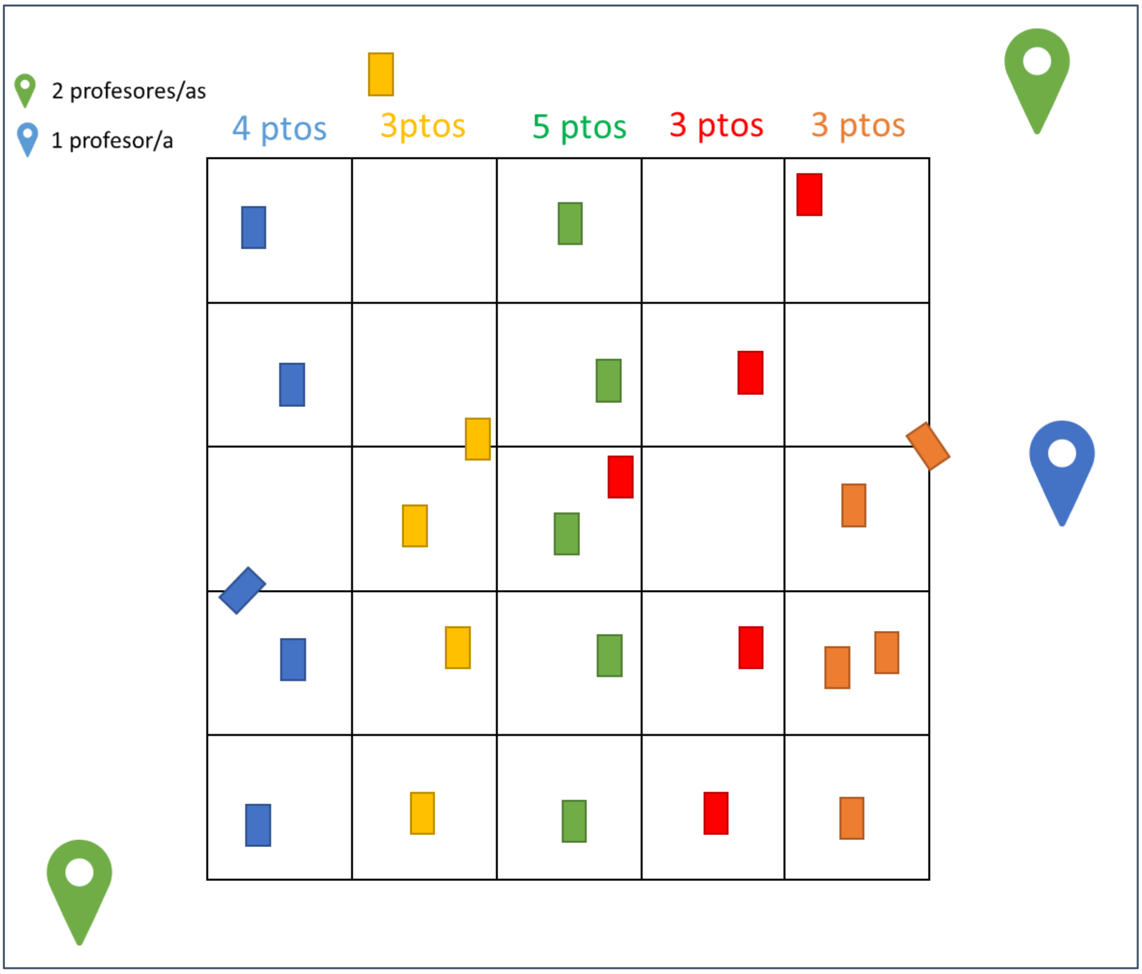

Ilustración 6 - Gráfico de ejemplos de la prueba 4: lanzamientos

Reglas e instrucciones para comunicar a los niños/as

- ✓ Objetivo: dejar un saco en cada cuadrado del propio carril.
- ✓ **Mano lanzadora:** primero lanzarán todos los niños/as del mismo grupo los cinco sacos con la mano derecha. Recogidos los sacos, se volverán a lanzar con la mano izquierda por parte del mismo grupo.
- ✓ **Estilo de lanzamiento**: **todos los sacos** se lanzarán de uno en uno, hacia arriba y con un movimiento de balanceo por debajo del hombro (estilo bolos-petanca).

- ✓ **Fin de la prueba**: cuando el niño/a lanza los cinco sacos y se ha registrado el resultado.
- ✓ Recomendación: que cada lanzador/a tenga sacos de diferente color que los que tienen justo al lado y que no los recojan hasta recibir el aviso.
- ✓ Punto clave 1: los sacos se lanzan de **uno en uno**, desde **detrás de la línea** y se puede apartar los que no he lanzado si me molestan para lanzar bien.
- ✓ Punto clave 2: todos los cuadrados tienen que quedar ocupados. Si un saco está entre dos cuadrados, no cuenta ni para uno ni para el otro.
- ✓ Punto clave 3: todos los cuadrados puntúan igual (**1 punto**). Elegir según esto a que cuadrado se va a lanzar.
- ✓ Punto clave 4: después de realizar la demostración poner ejemplos de los posibles fallos en la prueba. Explicar qué cosas no se pueden hacer.

Puntuación de la prueba y errores más comunes

- ✓ Puntuación: **se reciben tantos puntos como número de cuadrados del propio carril tengan al menos un saco dentro.**
- ✓ Error 1: lanzar con la otra mano. Hay que pedir que se repita el lanzamiento.
- ✓ Error 2: lanzar varios sacos a la vez. Hay que pedir que se repita el lanzamiento.
- ✓ Error 3: lanzar por encima del hombro. Hay que pedir que repita el lanzamiento.
- ✓ Error 4: que el saco no esté completamente dentro y tenga parte del peso en fuera de uno de los cuadrados. Si el peso del saco está dentro del cuadrado y únicamente sobre pasa la tela, será contado como válido.

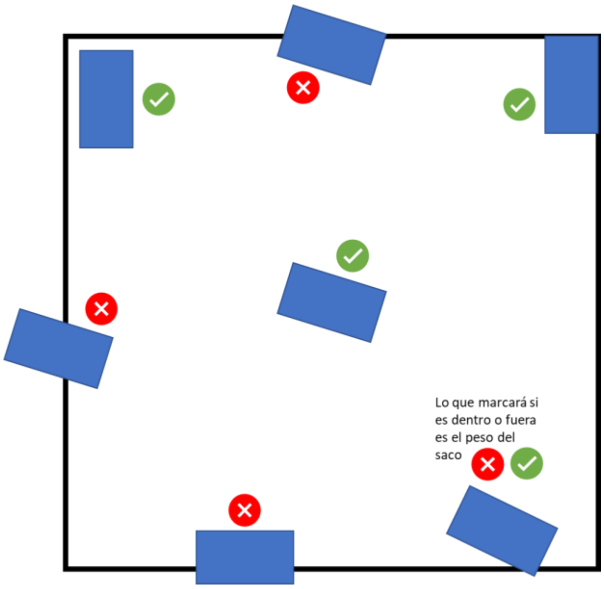

Ilustración 7 - Fallos en la zona de caída de los sacos

Prueba 5: Golpeo con el pie

La prueba de golpeo con el pie consiste en dar una patada a los 5 sacos de uno en uno intentando, dentro del propio carril, dejar cada uno de ellos en un cuadrado. Aquellos sacos que no estén completamente dentro de un cuadrado no contarán. Además, cada cuadrado tendrá una máxima puntuación de un saco, por tanto, si hay dos sacos solo contaría 1 punto. Está prueba solo se realizará con la pierna hábil.

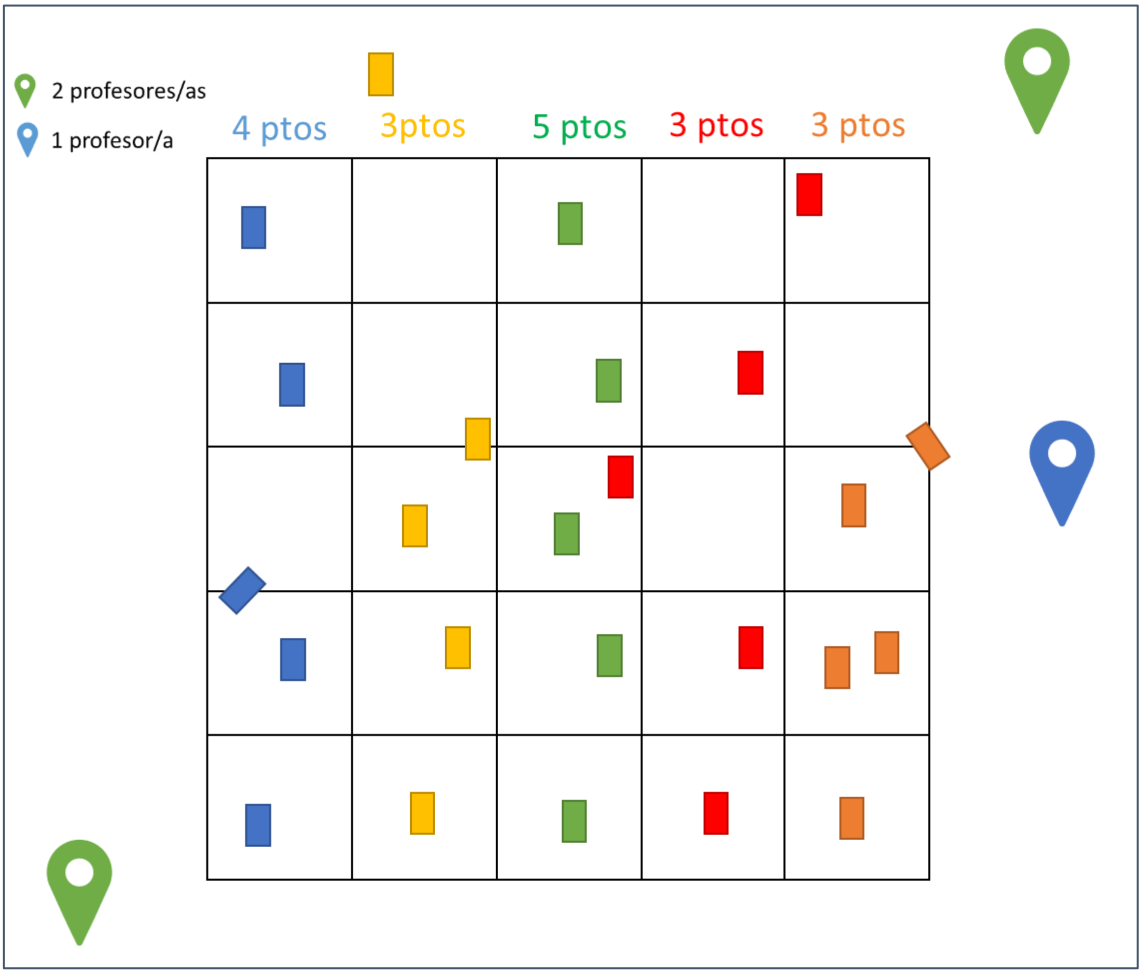

Ilustración 8 - Gráfico de ejemplos de la prueba 5: Golpeo con el pie

Reglas e instrucciones para comunicar a los niños/as

- ✓ Objetivo: dejar un saco en cada cuadrado del propio carril.
- ✓ **Pierna hábil:** el niño/a tendrá que elegir cuál es su pierna hábil de golpeo y solamente realizará la tarea con esa pierna. Los cinco golpeos deben ser con la misma pierna hábil.
- ✓ **Fin de la prueba**: cuando el niño/a golpea los cinco sacos y se ha registrado el resultado.

- ✓ Recomendación: que cada niño/a tenga sacos de diferente color que los que tienen justo al lado y que no los recojan hasta recibir el aviso.
- ✓ Punto clave 1: los sacos se golpean de uno en uno, desde detrás de la línea y se puede apartar los que no he golpeado aun si me molestan para golpear bien.
- ✓ Punto clave 2: todos los cuadrados tienen que quedar ocupados. Si un saco está entre dos cuadrados, no cuenta ni para uno ni para el otro.
- ✓ Punto clave 3: todos los cuadrados puntúan igual (1 punto). Elegir según esto a que cuadrado se va a intentar llegar con el golpeo.
- ✓ Punto clave 4: después de realizar la demostración poner ejemplos de los posibles fallos en la prueba. Explicar qué cosas no se pueden hacer.

Puntuación de la prueba y errores más comunes

- ✓ Puntuación: **se reciben tantos puntos como número de cuadrados del propio carril tengan al menos un saco dentro.**
- ✓ Error 1: golpear con la otra pierna. Hay que pedir que se repita el golpeo.
- ✓ Error 2: golpear varios sacos a la vez. Hay que pedir que se repita el golpeo.
- ✓ Error 3: **pisar el saco y lanzar arrastrando**. Hay que pedir que repita el golpeo.
- ✓ Error 4: que el saco no esté completamente dentro y tenga parte del peso en fuera de uno de los cuadrados. Si el peso del saco está dentro del cuadrado y únicamente sobre pasa la tela, será contado como válido.

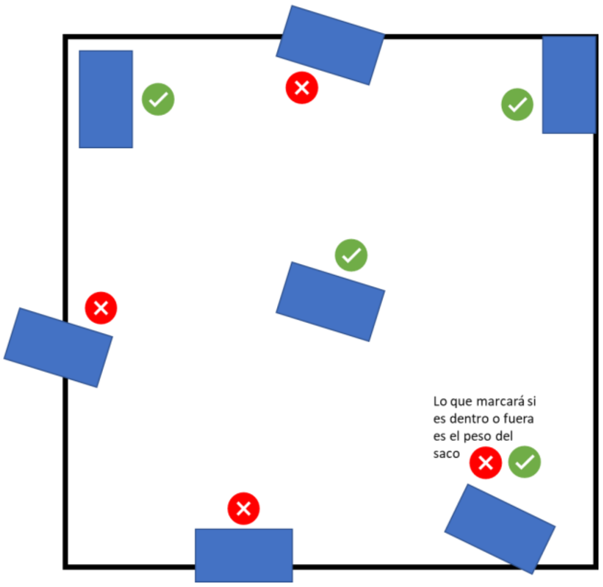

Ilustración 9 - Fallos en la zona de caída de los sacos

Prueba 6: Equilibrios

La prueba de equilibrios tiene 4 tareas diferentes que los niños/as deberán ir completando. Todos/as realizarán la primera tarea antes de pasar a la segunda. Estas tareas son:

- ✓ **Equilibrio 1:** consiste en dar **tres vueltas** con el saco alrededor del cuerpo, con los **pies juntos** sobre la primera línea.
- ✓ **Equilibrio 2:** consiste en dar **tres vueltas** con el saco alrededor del cuerpo, solamente apoyando **un pie** (“pata coja”) sobre la primera línea.
- ✓ **Equilibrio 3:** consiste en recoger un saco del suelo manteniéndose en equilibrio sobre una sola pierna. Para ello, el niño/a extenderá el brazo hasta que esté en paralelo al suelo, desde ahí **dejará caer el saco para posteriormente recogerlo**. Manteniendo en todo momento el equilibrio sobre una pierna.
- ✓ **Equilibrio 4:** consiste en dar tres vueltas con el saco alrededor del cuerpo, solamente apoyando un pie (“pata coja”) sobre la primera línea y manteniendo los ojos cerrados.

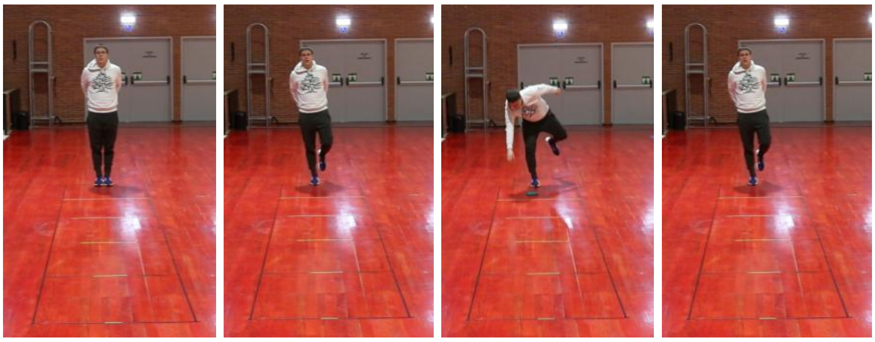

Ilustración 10 - Gráfico de ejemplos de la prueba 6: Equilibrios

Reglas e instrucciones para comunicar a los niños/as

- ✓ Objetivos: en las tareas 1,2 y 4, el objetivo es dar tres vueltas con el saco alrededor del cuerpo sin perder el equilibrio. En la tarea 3, el objetivo es: dejar caer el saco y recogerlo sin perder el equilibrio ni tocar el suelo.
- ✓ **Elección de la pierna de apoyo**: la pierna de apoyo es a elección del niño/a y no se puede cambiar durante el transcurso de cada una de las tareas, sí para una tarea diferente.
- ✓ **Fin de la prueba**: cuando cada uno de los niños/as termina su tarea.
- ✓ Punto clave 1: cada niño/a va a llevar un ritmo diferente. Fijarse en lo que hace la persona que está a su lado solamente dificulta la prueba.
- ✓ Punto clave 2: completar siempre las 3 vueltas en las tareas 1,2 y 4.

- ✓ Punto clave 3: demostrar insistentemente cómo se realiza la tarea 3 y corregir antes de que los niños/as empiecen a realizarla.
- ✓ Punto clave 4: después de realizar la demostración poner ejemplos de los posibles fallos en la prueba. Explicar qué cosas no se pueden hacer.

Puntuación de la prueba y errores más comunes

- ✓ Puntuación: cada una de las tareas recibirá una calificación de apto (sí) o no apto (no). Apto (sí) reflejará que el niño/a ha completado la tarea sin cometer ningún error. No apto (no) reflejará que el niño/a ha cometido un error durante la realización de la tarea.
- ✓ Errores de la tarea 3: en la tarea 3 puede existir además de errores de equilibrio, el error de que un niño/a se apoye en el suelo con la mano al ir a recoger el saco.
- ✓ Error de equilibrio: los errores de equilibrio son los mismos que en las pruebas de saltos, pero se retoman a continuación para que queden claros de nuevo.

Cuando en la prueba de equilibrios hablamos de perdida de equilibrio la situación más evidente es en la que el niño/a se caen al suelo, sin embargo, hay otras situaciones donde el niño/a pierde el equilibrio y su manifestación más clara es el arrastre del pie. Un ejemplo ilustrativo es pensar en el movimiento de un flan cuando "tiembla", que en el terreno de las HFM tendría la denominación de ajuste propioceptivo. Todos estos ajustes donde el niño/a **<u>no</u> desplaza su apoyo** serían **legales**. En el caso en el que el pie se **arrastre de su apoyo inicial, este se contaría como fallo.**

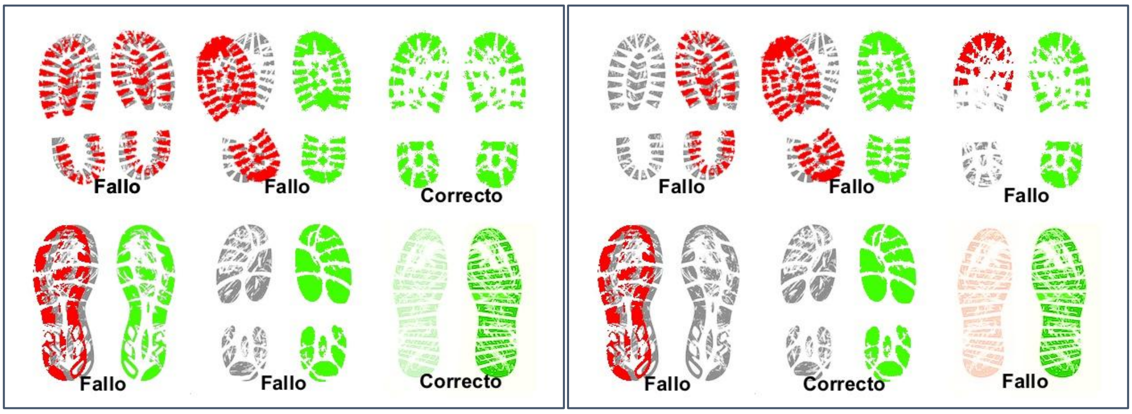

Ilustración 11 - Fallos provocados por la pérdida de equilibrio: ejemplos

Ejemplos de situaciones válidas:

- ✓ El pie de apoyo está fijo **sin arrastrarse** por el suelo y la huella que realiza el niño/a es fija.

- ✓ **La fuerza recae sobre el apoyo de un pie**, y este no se levanta nunca completamente del suelo.
- ✓ El pie de apoyo realiza un balanceo **sobre su propia huella** que termina estabilizándose.

Ejemplos de situaciones incorrectas o de pérdidas de equilibrio:

- ✓ Caerse.
- ✓ En los equilibrios sobre una pierna apoyar el otro pie parcial o completamente, en el suelo o sobre la otra pierna.
- ✓ El pie de apoyo se arrastra por el suelo modificando su posición inicial en la marca.
- ✓ El pie de apoyo se levanta de su posición inicial en la marca.

3. Recogida de datos

La hoja de puntuación del maestro/a es la que se va a utilizar para la recogida de datos. Está disponible tanto en formato PDF como en formato Excel. Cada maestro/a deberá elegir la manera que le resulte más cómoda para tomar registro de los datos sabiendo que hay que evaluar a varios niños/as al mismo tiempo. La recomendación es que se realice un registro en papel para poder después con tranquilidad hacer el paso al registro electrónico.

Con la hoja de puntuación del maestro/a se pueden preparar con anterioridad los grupos y llevarlos organizados a la evaluación. De esta manera el único objetivo durante la misma es tomar registro de las puntuaciones que obtienen los niños/as. En la siguiente ilustración se muestra un ejemplo ficticio de hoja de registro.

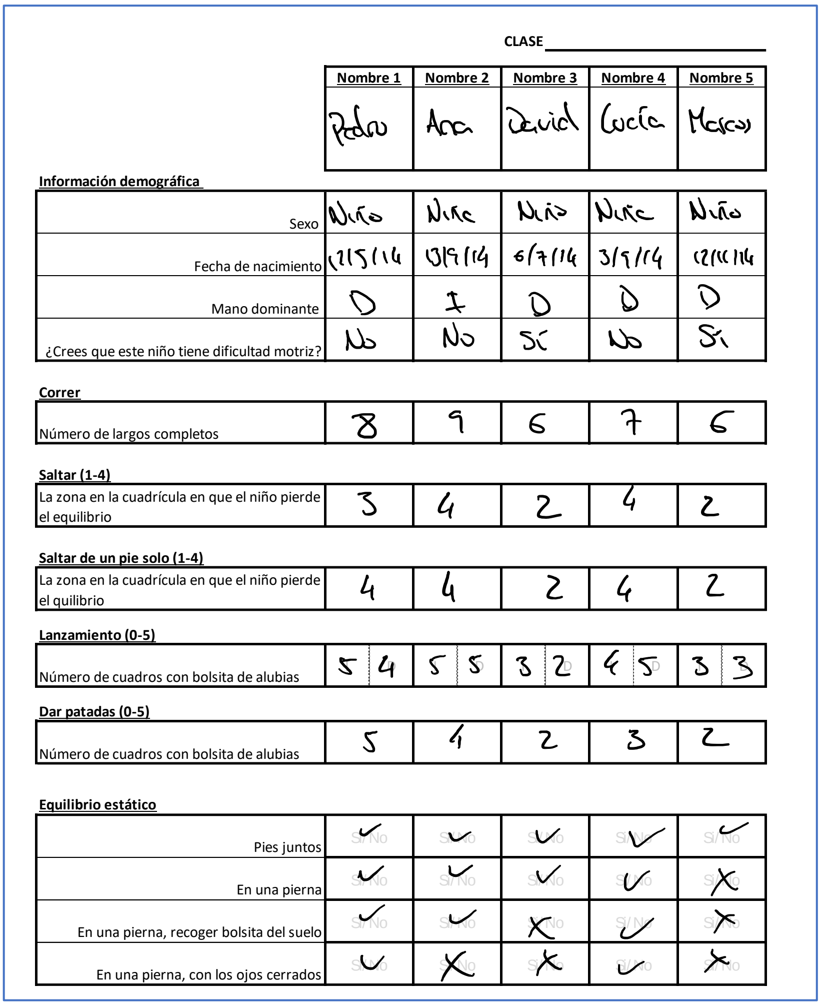

CLASE ____________

	Nombre 1	Nombre 2	Nombre 3	Nombre 4	Nombre 5
	Pedro	Ana	David	Lucía	Marcos
**Información demográfica**					
Sexo	Niño	Niña	Niño	Niña	Niño
Fecha de nacimiento	12/5/14	13/9/14	6/7/14	3/9/14	12/11/14
Mano dominante	D	I	D	D	D
¿Crees que este niño tiene dificultad motriz?	No	No	Sí	No	Sí
**Correr**					
Número de largos completos	8	9	6	7	6
**Saltar (1-4)**					
La zona en la cuadrícula en que el niño pierde el equilibrio	3	4	2	4	2
**Saltar de un pie solo (1-4)**					
La zona en la cuadrícula en que el niño pierde el quilibrio	4	4	2	4	2
**Lanzamiento (0-5)**					
Número de cuadros con bolsita de alubias	5 / 4	5 / 5	3 / 2	4 / 5	3 / 3
**Dar patadas (0-5)**					
Número de cuadros con bolsita de alubias	5	4	2	3	2
**Equilibrio estático**					
Pies juntos	Sí/No ✓	Sí/No ✓	Sí/No ✓	Sí/No ✓	Sí/No ✓
En una pierna	Sí/No ✓	Sí/No ✓	Sí/No ✓	Sí/No ✓	Sí/No ✗
En una pierna, recoger bolsita del suelo	Sí/No ✓	Sí/No ✓	Sí/No ✗	Sí/No ✓	Sí/No ✗
En una pierna, con los ojos cerrados	Sí/No ✓	Sí/No ✗	Sí/No ✗	Sí/No ✓	Sí/No ✗

Ilustración 12 - Hoja de puntuación del maestro/a
