# Supplementary material for: Validation of FUNMOVES: A reliable tool for assessing motor skills in Spanish schoolchildren
Source: PLoS One. 2025 Dec 5;20(12):e0337605. doi: 10.1371/journal.pone.0337605 (PMC12680221; doi:10.1371/journal.pone.0337605)
Supplement: S2 File — (PDF) [file pone.0337605.s015.pdf]

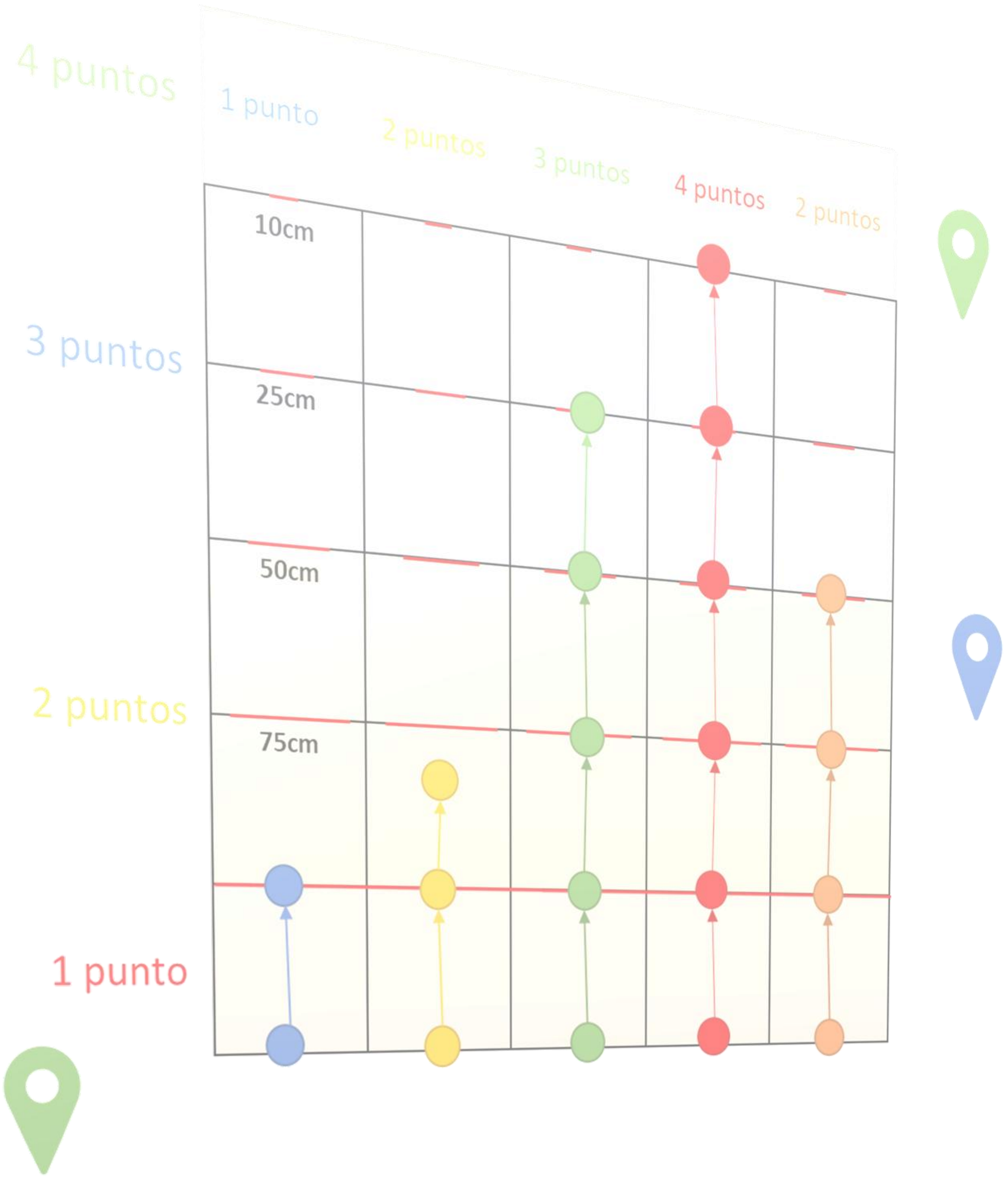

FUNMOVES – SPANISH VERSION

A USER GUIDE FOR SETTING UP, RUNNING, AND SCORING THE ACTIVITIES

Universidad de Navarra | FACULTAD DE EDUCACIÓN Y PSICOLOGÍA

UNIVERSITY OF LEEDS

I. Introduction ____ *2*

Aim of FUNMOVES ____ **2**

What is FUNMOVES? ____ **2**

What is FUNMOVES used for? ____ **3**

II. Phases ____ *4*

1. Setting up ____ **4**

Required resources ____ 4

Making groups ____ 4

Setting up the grid FUNMOVES ____ 5

Running FUNMOVES ____ 6

2. Activities ____ **7**

Activity 1: Running ____ 8

Activity 2: Jumping ____ 10

Activity 3: Hopping ____ 13

Activity 4: Throwing ____ 16

Activity 5: Kicking ____ 19

Activity 6: Balance ____ 21

3. Data collection ____ **24**

I. Introduction

This manual has been adapted by the 'Neurodevelopment and Learning' research group at the University of Navarra from the original FUNMOVES tool developed by the University of Leeds.

It is published under a 'Creative Commons Attribution' (CC BY) license, which allows unrestricted use, distribution, and adaptation, provided that the original source and the adaptation are properly cited.

Aim of FUNMOVES

The main goal of FUNMOVES is **to provide schools with an effective tool for assessing children's fundamental movement skills**. By identifying children who struggle with key motor skills, additional and personalized support can be offered to help them improve their abilities and reduce the risk of physical and mental health issues, as well as poor academic performance.

Moreover, FUNMOVES has been designed with school feasibility in mind, meaning that an entire class can be assessed within one hour during Physical Education lessons.

What is FUNMOVES?

FUNMOVES is an evidence-based assessment tool developed at the University of Leeds by the research team composed of Lucy Eddy, under the supervision of Liam Hill and Nick Preston, to identify potential difficulties in **fundamental movement skills (FMS)** in children. It specifically focuses on six key motor skills: running, jumping, hopping, throwing, kicking, and balancing. As a screening tool, the outcome of the FUNMOVES assessment serves as additional support to the information already available to the school and the family, providing a structured instrument to help detect potential difficulties in FMS.

The FUNMOVES assessments take place on a **5x5 meter grid**, where each student has their own lane measuring 1 meter wide by 1 meter long. During the assessment, five children participate simultaneously in the various motor skills mentioned earlier. The grid provides a standardized assessment space that ensures each child has enough room to perform the required skills. By assessing **five children at the same time**, the entire class can be evaluated within one hour during Physical Education lessons. This efficient

assessment method allows teachers to focus on developing individualized teaching plans for each child based on the assessment results.

Additionally, it is important to note that the **materials** used in the FUNMOVES assessment **are low-cost and commonly used in Physical Education** classes. This means that schools will not need to make a significant investment to use the test, as the necessary materials—such as beanbags, marking tape, and a stopwatch—are easily accessible and regularly used in PE lessons. This allows schools with different budgets to use FUNMOVES to identify their students' motor skills and provide the necessary additional support without incurring extra costs.

What is FUNMOVES used for?

Research has shown that children with low fundamental movement skills are at **greater risk** of experiencing adverse outcomes during childhood, such as **physical and mental health difficulties**, as well as poor academic performance. Identifying children who struggle with key motor skills will help schools effectively direct support to those students.

FUNMOVES therefore enables Physical Education teachers to collaborate with teachers of other subjects, as well as with the school's guidance department, to better understand the root of some children's difficulties in the classroom. As a result, the outcomes of this assessment will provide further insight into a child's performance and help identify potential difficulties that may be hindering their learning progress.

II. Phases

1. Setting up

Required resources

Grid

- ✓ 25 beanbags
- ✓ 60 m black marking tape
- ✓ 15 m color marking tape

Teacher folder

- ✓ Stopwatch
- ✓ Scoring sheets
- ✓ Pen
- ✓ Zone board + marker
- ✓ Support from another school staff member to help score the activities

Making groups

1. Divide your class into groups of up to five, based on the children's abilities

Groups can include up to five children, taking into account the challenge of observing all participants at once and the duration of the class. When forming the groups, consider each child's ability to run, jump, hop, throw, kick, and balance. These children will participate simultaneously, each in one of the grid lanes.

Grouping children with similar skill levels serves two purposes: First, it ensures that all children achieve similar results, reducing the likelihood of comparisons. Second, it prevents their performance from being influenced by their peers, meaning the child won't have immediate references from others who might perform significantly better or worse.

2. Complete the demographic information on the scoring sheet (for the teacher)

The dominant hand should be recorded as the hand the child uses to write. You should also indicate whether you believe a child: has motor difficulties, has trouble with handwriting, appears clumsy when moving around the classroom, or struggles to physically interact with objects.

CLASS ____________________

	Name 1	Name 2	Name 3	Name 4	Name 5

Demographics

Gender					
Date of Birth					
Dominant Hand					
Do you think this child has motor problems?					

Setting up the grid FUNMOVES

All activities take place in a 5x5 meter grid area, which must be set up using tape and a measuring tape according to the specifications shown below. It is important to note that the red lines in the diagram should be marked in a different color than the rest of the grid. Finally, make sure there is enough space in the classroom for the children who are not participating in the test at that moment to remain seated.

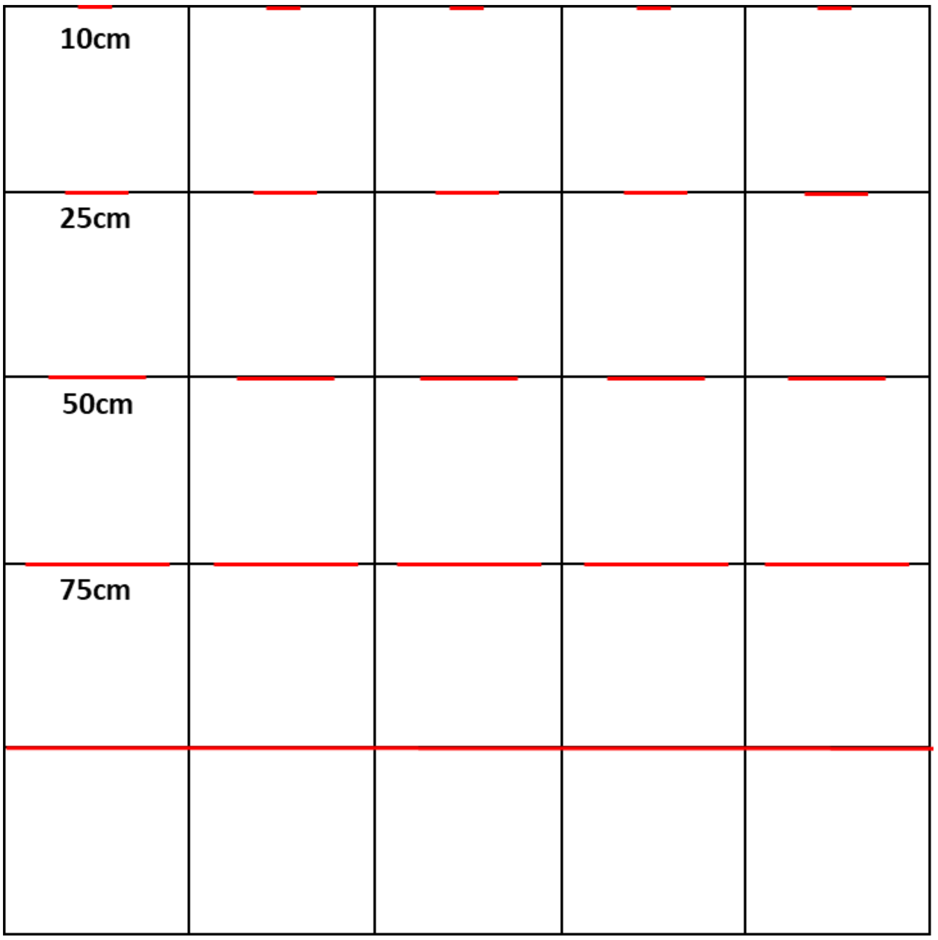

Running FUNMOVES

FUNMOVES can be implemented as an assessment test or carried out as a team competition to make it fun and engaging.

- ✓ During the test, ask the children who are not currently participating to wait on a bench.
- ✓ Carry out the activities one at a time, assessing all children before moving on to the next activity.
- ✓ Do not allow children to practice a task before performing it in the test.
- ✓ To prevent children from completing tasks incorrectly, tell them that their team will not earn any points if they cheat. They should not feel like they are being evaluated, but they should still try their best.
- ✓ Try not to make it obvious when a child makes a mistake or is "winning."
- ✓ Record failed attempts on the scoring sheet, but allow all children to continue and complete all activities, regardless of their performance level.
- ✓ Prepare the groups of five participants in advance and assign each child a specific lane.
- ✓ Bring the completed registration sheets with the personal information section filled out according to the assigned groups.
- ✓ Along with this manual, a PowerPoint presentation and explanatory videos for each test are available to visually complement the information provided here.

2. Activities

All the activities carried out in FUNMOVES take place within the space defined by the 5x5 meter grid. In this space, each student has their own lane to perform each of the tasks, with each marking corresponding to the specific requirements of the different tests..

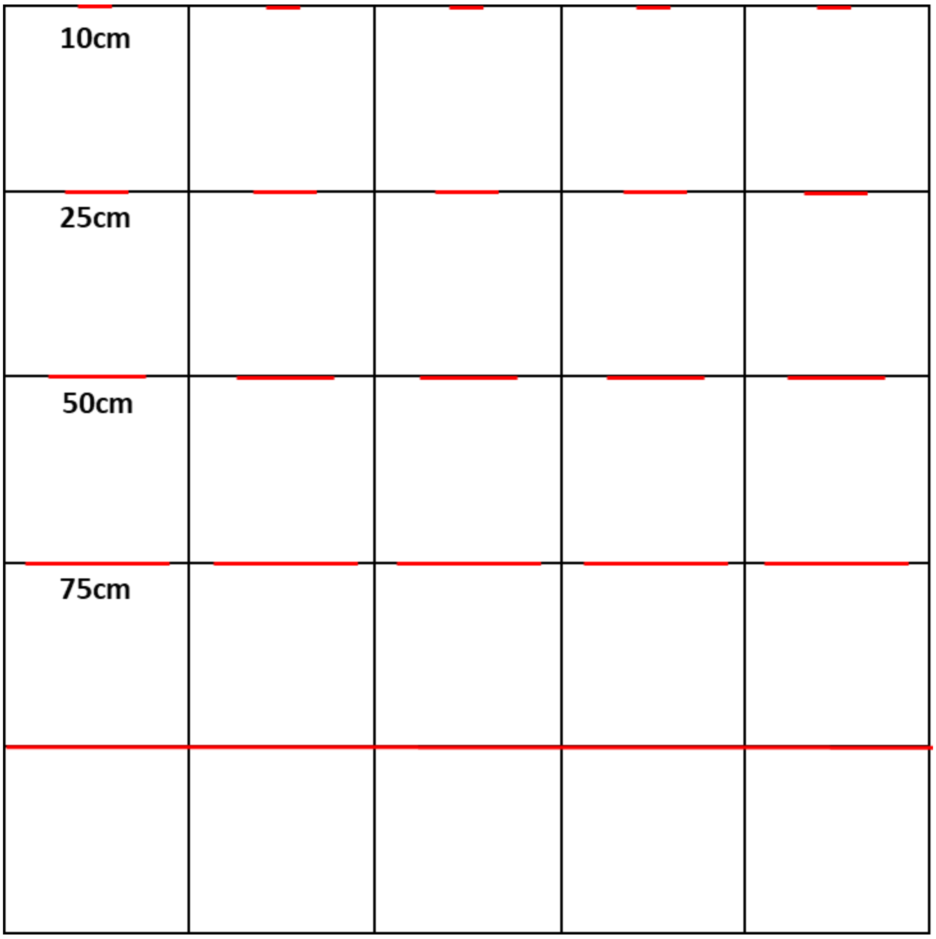

Activity 1: Running

The running test consists of covering the 5-meter distance as many times as possible within 15 seconds. To begin, the child stands with both feet on the line marking the start of their lane. Upon hearing the command '3, 2, 1, go,' they run to the far end of their lane, reaching the last line, and then return to the starting point. This process should be repeated as many times as possible until the command 'stop' is given. The score for this test is the number of complete lengths (5 meters) the child has completed.

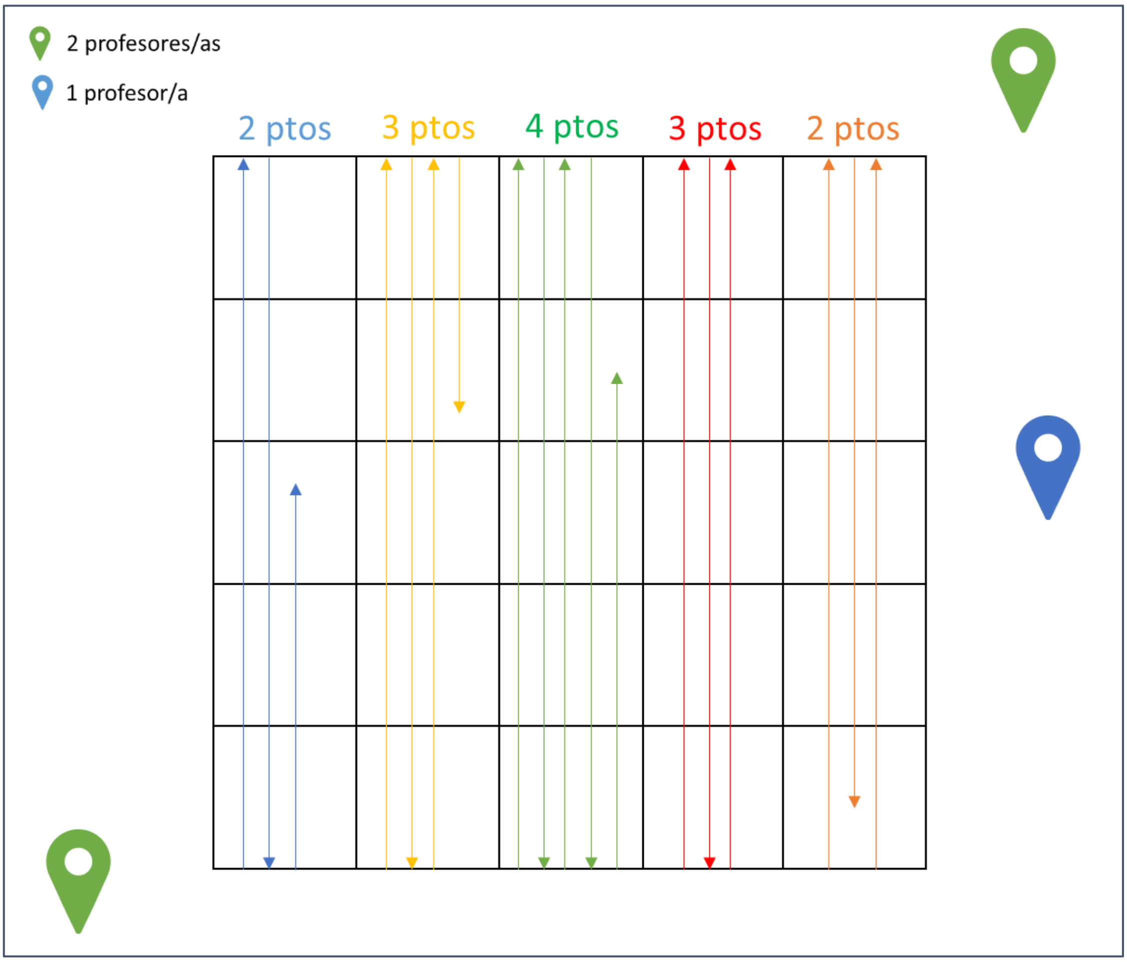

Fig 1 – Running scoring example

Rules and Instructions to Communicate to the Children

- ✓ Objective: Complete as many **lengths** as possible within the given time.
- ✓ Time limit: **15 seconds**.
- ✓ End of the test: Stop and sit down when you hear the word "**stop**," and wait until you are told to move.
- ✓ Key point 1: Run within your **own lane**. Notify if someone steps out.

- ✓ Key point 2: At both the start and end lines, and on each length, the child must **clearly step on the line** with at least one foot.
- ✓ Key point 3: Although sliding the foot is allowed, it is not an effective strategy as it wastes time.
- ✓ Key point 4: After demonstrating the activity, give examples of possible mistakes during the test. Explain what is not allowed.

Test Scoring and Common Errors

- ✓ Scoring: **Total number of complete lengths (5 meters).**
- ✓ Non-penalized error: **Stepping out of the lane**. The child should be reminded to stay in their own lane.
- ✓ Penalized error: **Not stepping on the line** when completing a length. That length will not count toward the final score. The child should be informed that the line was not stepped on.
- ✓ Penalized error: **Not making a proper change of direction** at the end of the lane, but instead turning in a small curve. The child should be informed that a proper change of direction was not made.

Activity 2: Jumping

The jumping activity consists of making small jumps to reach the next landing mark. Once there, the child must pause without losing balance and without moving their feet, waiting for the signal to start or continue. This process is repeated successively at each mark along their lane. In this test, points are not awarded for speed but for maintaining balance throughout the activity. Depending on the zone where the child loses balance, they will receive a different number of points.

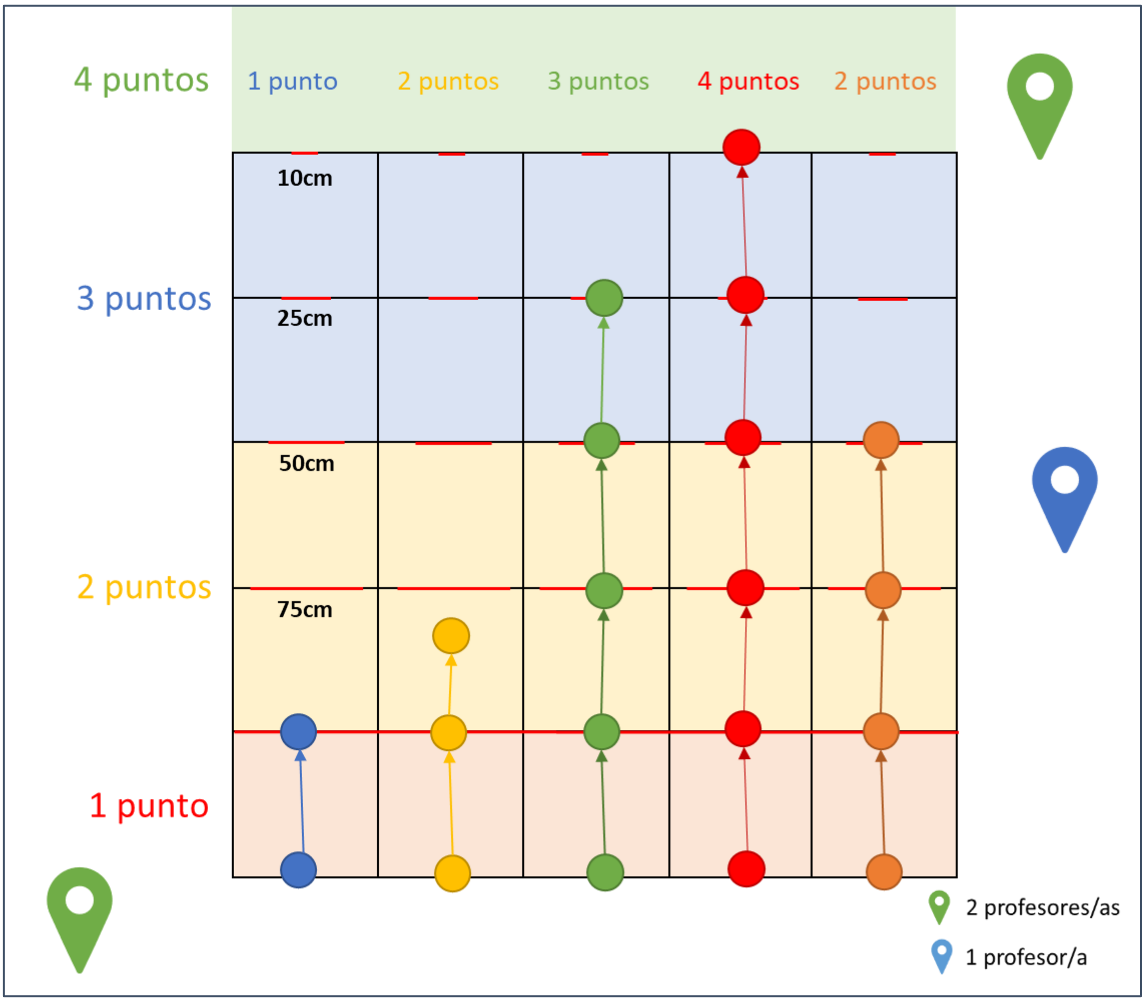

Fig 2 – Jumping scoring example

Rules and Instructions to Communicate to the Children

- ✓ Objective: **Move forward with small jumps**, pausing at each mark while maintaining balance.
- ✓ **Pause zones**: At each mark, children must wait for their classmates to arrive without losing balance and should not move to the next mark until the teacher says "1, 2, 3."

- ✓ **End of the test**: When the child holds the final pause for 3 seconds. If a child loses balance, record the incident but allow them to finish without drawing attention to it.
- ✓ Key point 1: The marks become narrower each time, so children must **focus on their own lane** to land correctly.
- ✓ Key point 2: Jumps should be **small and with both feet together**, aiming to occupy as much space as possible within the landing mark.
- ✓ Key point 3: Perform all pauses (including the final one) correctly without losing balance.
- ✓ Key point 4: After demonstrating the activity, give examples of possible mistakes during the test. Explain what is not allowed.

Test Scoring and Common Errors

- ✓ Scoring: Based on the **zone where the child loses balance (see previous illustration)**. If the child reaches the end without losing balance, they receive the maximum score (4 points). If balance is lost on a dividing line between zones, the score from the previous zone will be counted.
- ✓ Error 1: Making **only one jump** between each mark.
- ✓ Error 2: Placing **a foot outside the mark** when there is still space within it.
- ✓ Error 3: Not performing the **pause** correctly, or pausing in the middle of the lane.
- ✓ Error 4: **Losing balance**. The situations that are considered as loss of balance are explained below the diagram.

When we talk about loss of balance in the two-foot jumping test, the most obvious situation is when the child **falls** to the ground. However, there are other cases where balance is lost, and the clearest sign is **foot dragging**. A helpful example is to imagine the movement of a wobbly jelly—this would be referred to in the context of FMS as a proprioceptive adjustment. All of these adjustments, where the child <u>**does not**</u> **shift their point of support, are considered valid**. However, if the foot drags from its original position, it is counted as a fault.

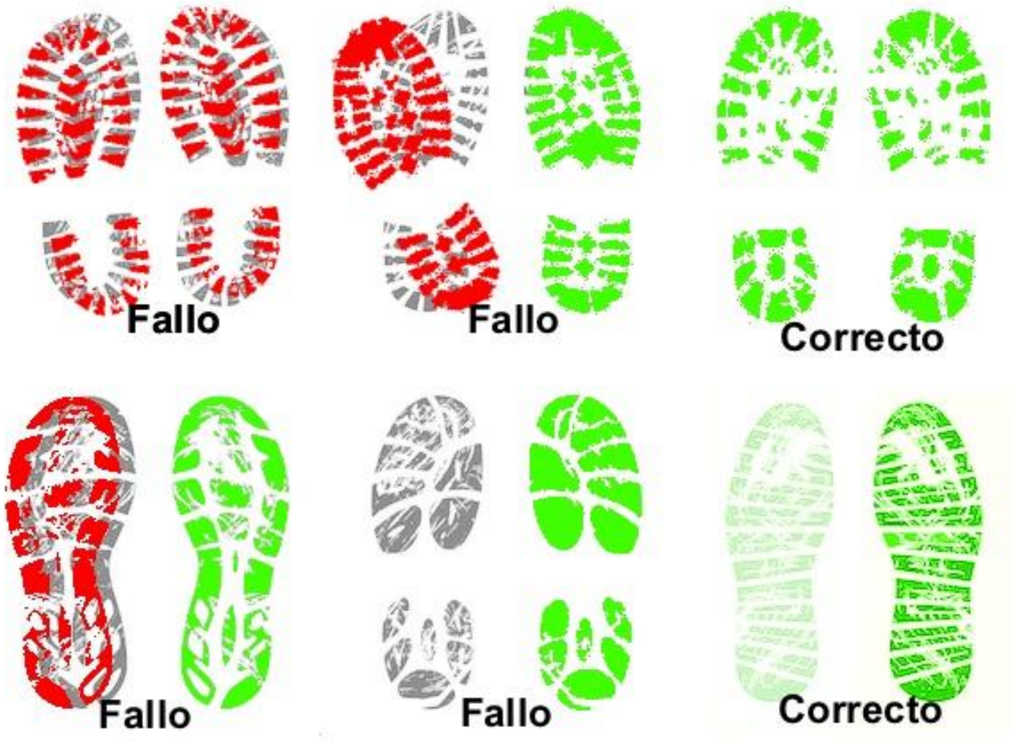

Fig 3 – Errors in balance

Examples of Valid Situations:

- ✓ Both feet remain fixed **without dragging** on the ground, and the footprint made by the child stays in place.
- ✓ **The weight is supported mainly on one foot**, but the other foot never lifts off the ground.
- ✓ The feet sway slightly **within their own footprint** but eventually stabilize.

Examples of Incorrect Situations or Loss of Balance:

- ✓ **Falling**.
- ✓ One or both **feet drag** on the ground, changing their initial position on the mark.
- ✓ One or both **feet lift** from their initial position on the mark.

Activity 3: Hopping

The hopping activity is similar to activity 2, with the only difference being that it is performed using one foot. It consists of making small hops to reach the next landing mark. Once there, the child must pause without losing balance and without moving their foot, waiting for the signal to start or continue. This process is repeated successively at each mark along their lane. In this test, points are not awarded for speed but for maintaining balance throughout the activity. Depending on the zone where the child loses balance, they will receive a different number of points.

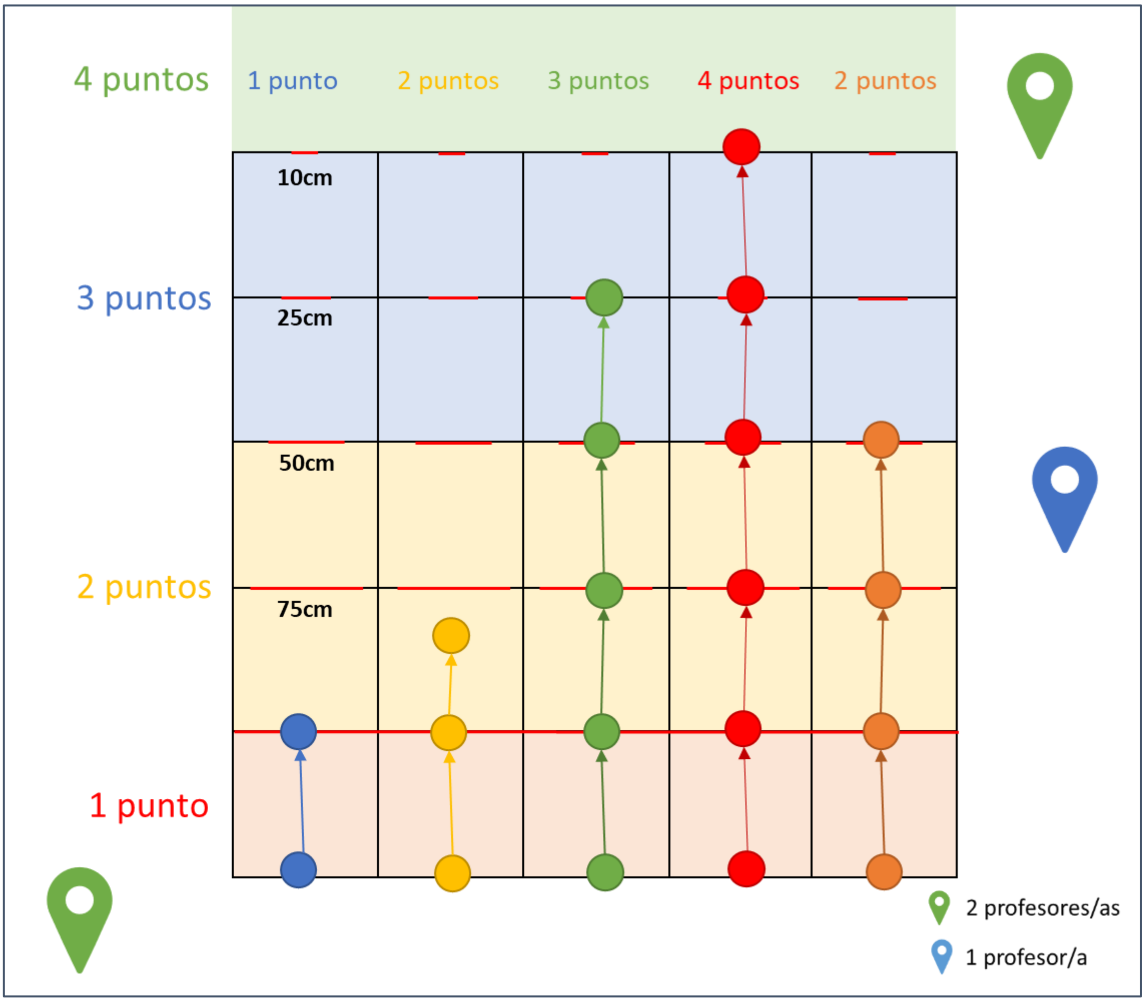

Fig 4 – Hopping scoring example

Rules and Instructions to Communicate to the Children

- ✓ Objective: To advance with **small hops on one leg**, pausing at each marked spot while maintaining balance.
- ✓ **Choice of supporting leg**: The child may choose which leg to use for support, and this choice must remain consistent throughout the test.

- ✓ **Pause zones**: At each marked spot, children must wait for their peers to arrive, maintaining balance and not proceeding to the next mark until the teacher says, "1, 2, 3."
- ✓ **End of the test**: The test concludes when the child holds the final position for 3 seconds. If a child loses balance, this should be recorded, but the child should be allowed to finish without drawing attention to the error.
- ✓ Key Point 1: The marks become progressively narrower, so each child must **focus on their designated lane** to ensure proper foot placement.
- ✓ Key Point 2: **Hops must be small and performed with one foot only**, aiming to occupy as much space as possible within the landing mark.
- ✓ Key Point 3: **Pauses**—including the final one—must be executed correctly without losing balance.
- ✓ Key Point 4: After the demonstration, provide examples of potential errors during the test and explain which actions are not permitted.

Test Scoring and Common Errors

- ✓ Scoring: **The score is determined by the zone in which the child loses balance**. If the child completes the entire course without losing balance, they receive the maximum score (4 points). If balance is lost on a dividing line between zones, the score corresponds to the previous zone.
- ✓ Error 1: Performing **only one hop** between marks.
- ✓ Error 2: Placing the **foot outside the mark** when there is sufficient space within it.
- ✓ Error 3: **Failing to perform the pause** correctly, or pausing in the middle of the course.
- ✓ Error 4: **Losing balance**. The specific situations that are considered as loss of balance are detailed below the diagram.

In the hopping activity, the most evident case of **loss of balance** is when the child falls to the ground. However, there are other situations that also indicate a loss of balance, the most common being foot **dragging**. A helpful analogy is the movement of a trembling flan, which in the context of FMS would be referred to as a proprioceptive adjustment. All of these adjustments, where the child <u>**does not**</u> **shift their point of support, are considered valid**. However, if the foot drags from its original position, it is counted as a fault.

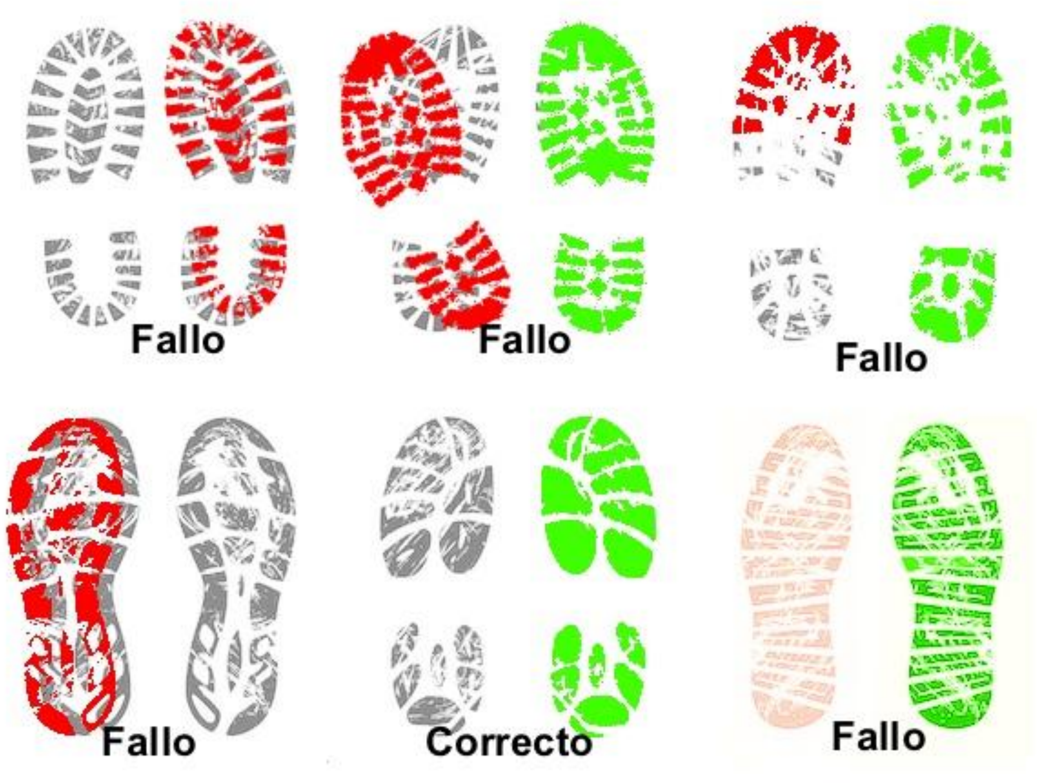

Fig 5 – Balance errors in hopping

Examples of Valid Situations:

- ✓ The supporting foot remains fixed without dragging along the ground, and the footprint made by the child stays in place.
- ✓ **The body weight is supported entirely by one foot**, which never fully lifts off the ground.
- ✓ The supporting foot may sway slightly **within its own footprint** before stabilizing.

Examples of Incorrect Situations or Loss of Balance:

- ✓ **Falling**.
- ✓ **Placing the other foot**—partially or fully—on the ground or against the supporting leg.
- ✓ **Dragging** the supporting foot along the ground, altering its initial position on the mark.
- ✓ **Lifting** the supporting foot completely from its initial position on the mark.

Activity 4: Throwing

The activity consists of throwing 5 beanbags, one at a time, aiming to land each one inside a square within the designated lane. Only beanbags that are entirely within a square will be counted. Additionally, each square can score a maximum of one point; therefore, if two beanbags land in the same square, only one point will be awarded. This test must be performed using both the right and left hand.

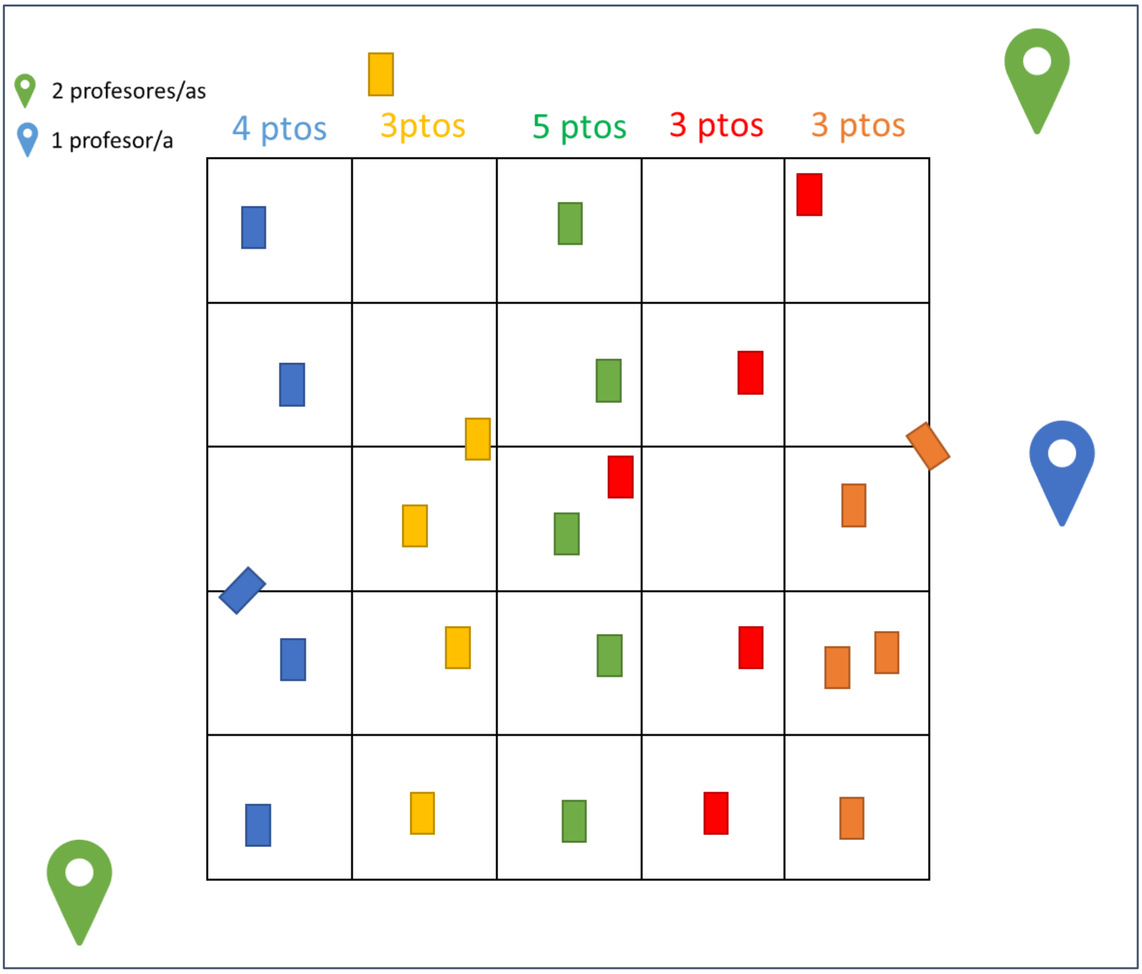

Fig 6 – Throwing scoring example

Rules and Instructions to Communicate to the Children

- ✓ Objective: To land one beanbag in each square within the designated lane.
- ✓ **Throwing hand**: All children in the same group will first throw the five beanbags using their right hand. Once the beanbags are collected, the same group will repeat the test using their left hand.
- ✓ **Throwing style**: All beanbags must be thrown one at a time, in an upward motion with an underhand swing (similar to bowling style).

- ✓ **End of the test**: When the child has thrown all five beanbags and the results have been recorded.
- ✓ Recommendation: Each thrower should use beanbags of a different color than those of the adjacent participants, and should not collect them until instructed to do so.
- ✓ Key Point 1: Beanbags must be thrown one at a time, **from behind the line**. Unused beanbags may be moved aside if they interfere with the throw.
- ✓ Key Point 2: All squares must be occupied. If a beanbag lands between two squares, it does not count for either.
- ✓ Key Point 3: All squares have the same value (**1 point**). Choose the target square accordingly.
- ✓ Key Point 4: After the demonstration, provide examples of possible errors during the test and explain which actions are not allowed.

Test Scoring and Common Errors

- ✓ Scoring: **The score corresponds to the number of squares within the participant's lane that contain at least one beanbag**.
- ✓ Error 1: Throwing with the opposite hand. The throw must be repeated.
- ✓ Error 2: Throwing multiple beanbags at once. The throw must be repeated.
- ✓ Error 3: Throwing overhand. The throw must be repeated.
- ✓ Error 4: The beanbag is not entirely within the square and part of its weight lies outside. If the weight of the beanbag is inside the square and only the fabric extends beyond the boundary, it will be considered valid.

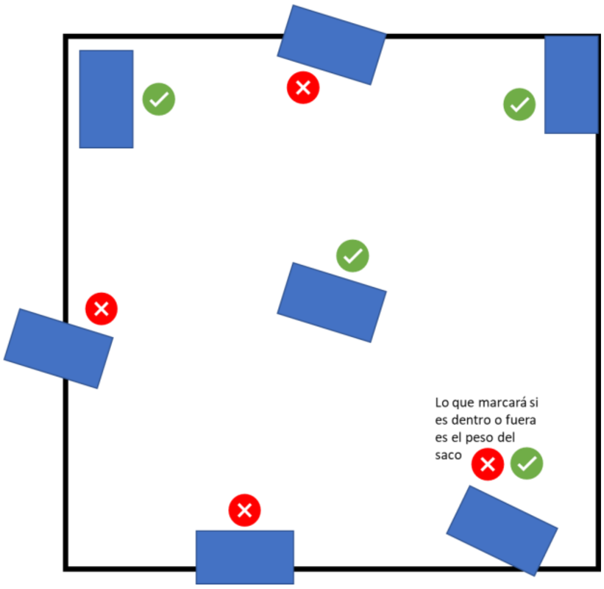

Fig 7 – Errors with the beanbag

Activity 5: Kicking

The activity consists of kicking 5 beanbags, one at a time, aiming to land each one inside a square within the designated lane. Only beanbags that are entirely within a square will be counted. Additionally, each square can score a maximum of one point; therefore, if two beanbags land in the same square, only one point will be awarded. This test must be performed using only the dominant leg.

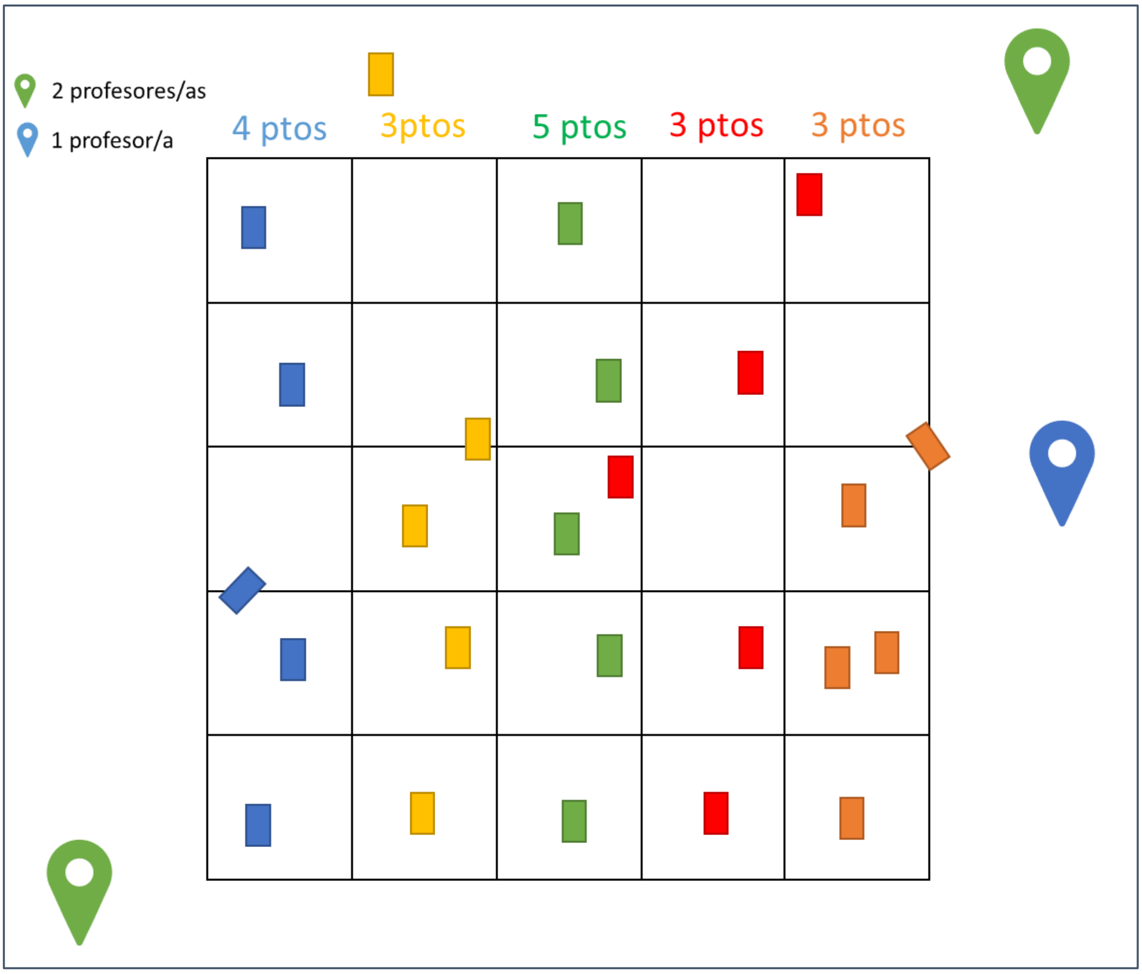

Fig 8 – Kicking scoring example

Rules and Instructions to Communicate to the Children

- ✓ Objective: To land one beanbag in each square within the designated lane.
- ✓ **Dominant leg**: The child must choose their dominant kicking leg and perform the entire task using only that leg. All five kicks must be executed with the same dominant leg.
- ✓ **End of the test**: When the child has kicked all five beanbags and the results have been recorded.

- ✓ Recommendation: Each child should use beanbags of a different color than those of the adjacent participants, and should not collect them until instructed to do so.
- ✓ Key Point 1: Beanbags must be kicked one at a time, from behind the line. Unused beanbags may be moved aside if they interfere with the kick.
- ✓ Key Point 2: All squares must be occupied. If a beanbag lands between two squares, it does not count for either.
- ✓ Key Point 3: All squares have the same value (1 point). Choose the target square accordingly.
- ✓ Key Point 4: After the demonstration, provide examples of possible errors during the test and explain which actions are not allowed.

Test Scoring and Common Errors

- ✓ Scoring: **The score corresponds to the number of squares within the participant's lane that contain at least one beanbag**.
- ✓ Error 1: Kicking with the opposite leg. The kick must be repeated.
- ✓ Error 2: Kicking multiple beanbags at once. The kick must be repeated.
- ✓ Error 3: **Stepping on the beanbag and sliding it instead of kicking**. The kick must be repeated.
- ✓ Error 4: The beanbag is not entirely within the square and part of its weight lies outside. If the weight of the beanbag is inside the square and only the fabric extends beyond the boundary, it will be considered valid.

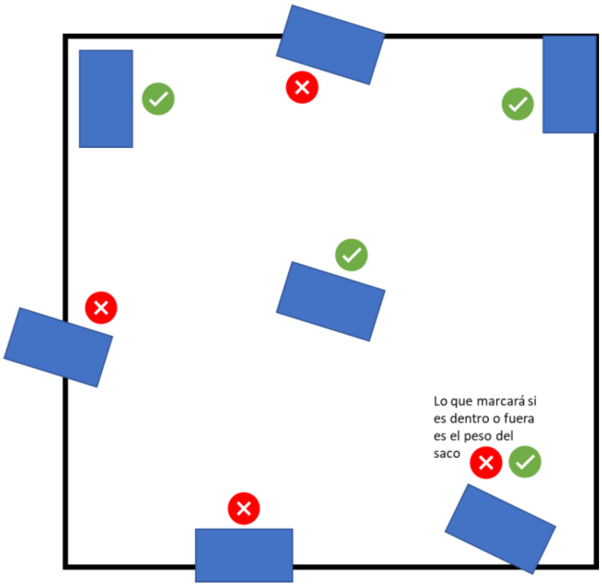

Fig 9 – Errors with the beanbag

Activity 6: Balance

The test consists of four different tasks that children must complete sequentially. All participants will complete the first task before moving on to the second, and so on. These tasks are:

- ✓ **Balance Task 1**: The child must perform three rotations of the beanbag around their body while standing with **both feet together** on the first line.
- ✓ **Balance Task 2**: The child must perform three rotations of the beanbag around their body while standing on **one foot** ("hopping position") on the first line.
- ✓ **Balance Task 3**: The child must pick up a beanbag from the ground while maintaining balance on one leg. To do this, the child extends their arm until it is parallel to the ground, **drops the beanbag from that position, and then picks it up**—maintaining balance on one leg throughout the task.
- ✓ **Balance Task 4**: The child must perform three rotations of the beanbag around their body while standing on one foot ("hopping position") on the first line, with their **eyes closed**.

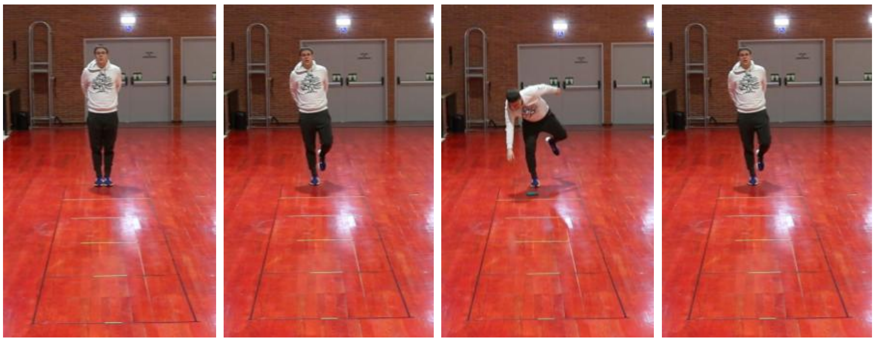

Fig 10 – Balance tasks examples

Rules and Instructions to Communicate to the Children

- ✓ Objectives: In Tasks 1, 2, and 4, the objective is to complete three rotations of the beanbag around the body without losing balance. In Task 3, the objective is to drop and pick up the beanbag without losing balance or touching the ground.
- ✓ **Choice of supporting leg**: The child may choose which leg to use for support, and this choice must remain the same throughout each individual task. A different leg may be chosen for a different task.
- ✓ **End of the test**: When each child completes their assigned task.
- ✓ Key Point 1: Each child will move at their own pace. Focusing on what the person next to them is doing will only make the task more difficult.

- ✓ Key Point 2: Always complete all three rotations in Tasks 1, 2, and 4.
- ✓ Key Point 3: Clearly and repeatedly demonstrate how to perform Task 3, and correct any misunderstandings before children begin.
- ✓ Key Point 4: After the demonstration, provide examples of possible errors during the test and explain which actions are not allowed.

Test Scoring and Common Errors

- ✓ Scoring: Each task will be graded as either Pass (Yes) or Fail (No). A Pass (Yes) indicates that the child completed the task without making any errors. A Fail (No) indicates that the child made an error during the execution of the task.
- ✓ Errors in Task 3: In addition to balance-related errors, Task 3 may include the specific error of a child using their hand to support themselves on the ground while picking up the beanbag.
- ✓ Balance Errors: Balance errors are the same as those defined in the hopping tests, but are reiterated below for clarity.

The most evident case of loss of balance is when the child falls to the ground. However, there are other situations that also indicate a loss of balance, the most common being foot dragging. A helpful analogy is the movement of a trembling flan, which in the context of Fundamental Motor Skills (FMS) is referred to as a proprioceptive adjustment. All of these adjustments, where the child **does not** **shift their point of support, are considered valid**. However, if the foot drags from its original position, it is counted as a fault**.**

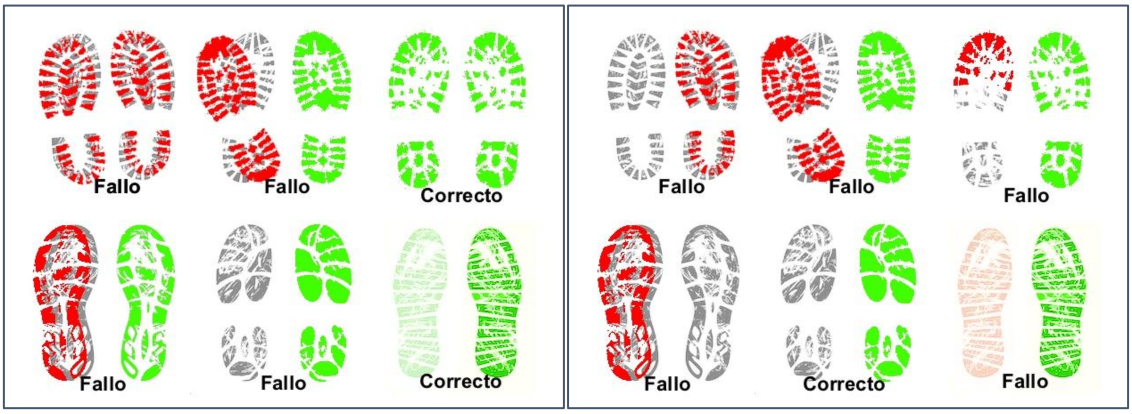

Fig 11 – Errors in loosing balance

Examples of Valid Situations:

- ✓ The supporting foot remains fixed without dragging along the ground, and the footprint made by the child stays in place.

- ✓ **The body weight is supported entirely by one foot**, which never fully lifts off the ground.
- ✓ The supporting foot may sway slightly within its own footprint before stabilizing.

Examples of Incorrect Situations or Loss of Balance:

- ✓ Falling.
- ✓ In single-leg balance tasks, placing the other foot—partially or fully—on the ground or against the supporting leg.
- ✓ Dragging the supporting foot along the ground, altering its initial position on the mark.
- ✓ Lifting the supporting foot from its initial position on the mark.

3. Data collection

The teacher's scoring sheet will be used for data collection. It is available in both PDF and Excel formats. Each teacher may choose the format that is most convenient for recording data, keeping in mind that multiple children will be evaluated simultaneously. It is recommended to use a paper-based record during the evaluation, which can later be transferred calmly to the digital version.

The teacher's scoring sheet also allows for groups to be prepared in advance and brought to the evaluation in an organized manner. This way, the only focus during the assessment is to record the scores obtained by the children. The following illustration shows a fictional example of a scoring sheet.

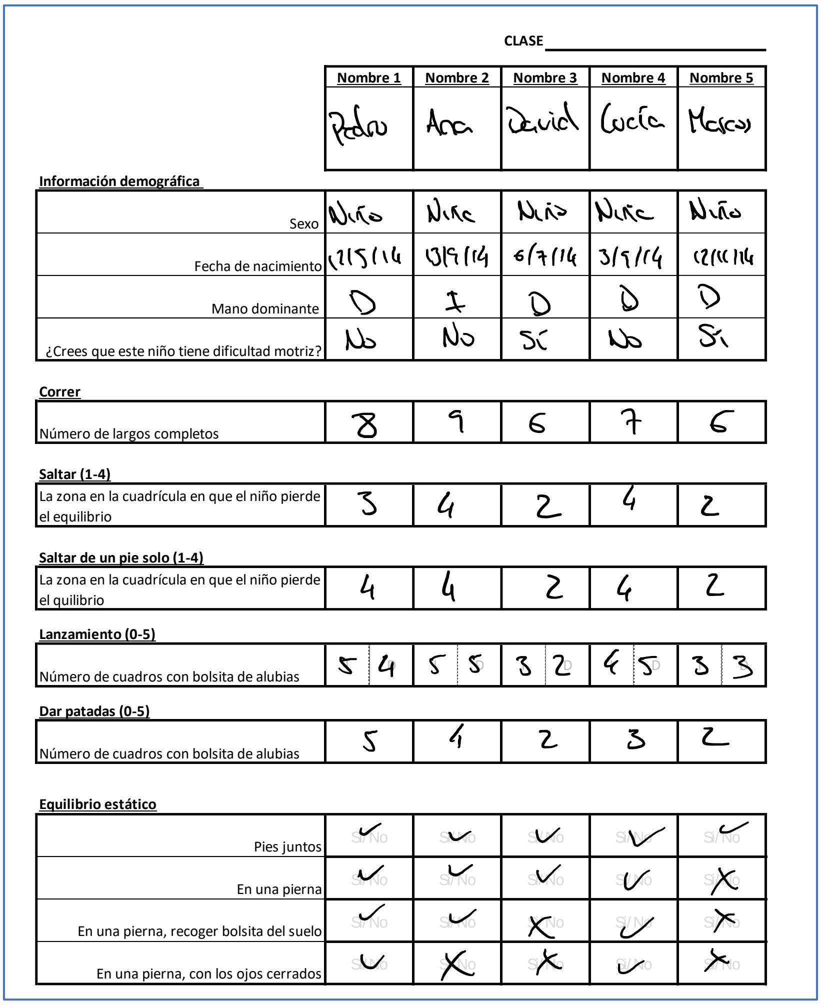

CLASE ____________

	Nombre 1	Nombre 2	Nombre 3	Nombre 4	Nombre 5
	Pedro	Ana	David	Lucía	Marcos
**Información demográfica**					
Sexo	Niño	Niña	Niño	Niña	Niño
Fecha de nacimiento	12/5/14	13/9/14	6/7/14	3/9/14	12/11/14
Mano dominante	D	I	D	D	D
¿Crees que este niño tiene dificultad motriz?	No	No	Sí	No	Sí
**Correr**					
Número de largos completos	8	9	6	7	6
**Saltar (1-4)**					
La zona en la cuadrícula en que el niño pierde el equilibrio	3	4	2	4	2
**Saltar de un pie solo (1-4)**					
La zona en la cuadrícula en que el niño pierde el quilibrio	4	4	2	4	2
**Lanzamiento (0-5)**					
Número de cuadros con bolsita de alubias	5 / 4	5 / 5	3 / 2	4 / 5	3 / 3
**Dar patadas (0-5)**					
Número de cuadros con bolsita de alubias	5	4	2	3	2
**Equilibrio estático**					
Pies juntos	Sí / No ✓	Sí / No ✓	Sí / No ✓	Sí / No ✓	Sí / No ✓
En una pierna	Sí / No ✓	Sí / No ✓	Sí / No ✓	Sí / No ✓	Sí / No ✗
En una pierna, recoger bolsita del suelo	Sí / No ✓	Sí / No ✓	Sí / No ✗	Sí / No ✓	Sí / No ✗
En una pierna, con los ojos cerrados	Sí / No ✓	Sí / No ✗	Sí / No ✗	Sí / No ✓	Sí / No ✗

Fig 12 – Teacher scoring sheet
