# Supplementary material for: Validation of FUNMOVES: A reliable tool for assessing motor skills in Spanish schoolchildren
Source: PLoS One. 2025 Dec 5;20(12):e0337605. doi: 10.1371/journal.pone.0337605 (PMC12680221; doi:10.1371/journal.pone.0337605)
Supplement: S3 File — (PDF) [file pone.0337605.s016.pdf]

CLASE ____________________

	Nombre 1	Nombre 2	Nombre 3	Nombre 4	Nombre 5

Información demográfica

Sexo					
Fecha de nacimiento					
Mano dominante					
¿Crees que este niño tiene dificultad motriz?					

Correr

Número de largos completos					

Saltar (1-4)

La zona en la cuadrícula en que el niño pierde el equilibrio					

Saltar de un pie solo (1-4)

La zona en la cuadrícula en que el niño pierde el quilibrio					

Lanzamiento (0-5)

Número de cuadros con bolsita de alubias	I	D	I	D	I	D	I	D	I	D

Dar patadas (0-5)

Número de cuadros con bolsita de alubias					

Equilibrio estático

Pies juntos	Si/No	Si/No	Si/No	Si/No	Si/No
En una pierna	Si/No	Si/No	Si/No	Si/No	Si/No
En una pierna, recoger bolsita del suelo	Si/No	Si/No	Si/No	Si/No	Si/No
En una pierna, con los ojos cerrados	Si/No	Si/No	Si/No	Si/No	Si/No
