# Supplementary material for: Validation of FUNMOVES: A reliable tool for assessing motor skills in Spanish schoolchildren
Source: PLoS One. 2025 Dec 5;20(12):e0337605. doi: 10.1371/journal.pone.0337605 (PMC12680221; doi:10.1371/journal.pone.0337605)
Supplement: S4 File — (PDF) [file pone.0337605.s017.pdf]

CLASS ____________________

	Name 1	Name 2	Name 3	Name 4	Name 5

Demographics

Gender					
Date of Birth					
Dominant Hand					
Do you think this child has motor problems?					

Running

Number of Full Lengths					

Jumping (1-4)

Zone on the grid where the child lost balance					

Hopping (1-4)

Zone on the grid where the child lost balance					

Throwing (0-5)

Number of boxes with beanbags in	L | R	L | R	L | R	L | R	L | R

Kicking (0-5)

Number of boxes with beanbags in					

Static Balance

Legs Together	y/ N	Y/ N	Y/ N	Y/ N	Y/ N
One Leg	Y/ N	Y/ N	Y/ N	Y/ N	Y/ N
Beanbag on the Floor One Leg	Y/ N	Y/ N	Y/ N	Y/ N	Y/ N
One Leg Eyes Closed	Y/ N	Y/ N	Y/ N	Y/ N	Y/ N
